# 4-Alkyl-4*H*-thieno[2′,3′:4,5]pyrrolo[2,3-*b*]quinoxaline Derivatives as New Heterocyclic Analogues of Indolo[2,3-*b*]quinoxalines: Synthesis and Antitubercular Activity

**DOI:** 10.3390/ijms26010369

**Published:** 2025-01-03

**Authors:** Gusein A. Sadykhov, Danila V. Belyaev, Ekaterina E. Khramtsova, Diana V. Vakhrusheva, Svetlana Yu. Krasnoborova, Dmitry V. Dianov, Marina G. Pervova, Gennady L. Rusinov, Egor V. Verbitskiy, Valery N. Charushin

**Affiliations:** 1 Postovsky Institute of Organic Synthesis, Ural Branch of the Russian Academy of Sciences, S. Kovalevskoy Street, 22, Ekaterinburg 620137, Russiad734698@yandex.ru (D.V.B.); pervova@ios.uran.ru (M.G.P.); rusinov@ios.uran.ru (G.L.R.); valery-charushin-562@yandex.ru (V.N.C.); 2 Department of Organic and Biomolecular Chemistry, Ural Federal University, Mira Street, 19, Ekaterinburg 620002, Russia; 3 Ural Research Institute for Phthisiopulmonology—The Branch of National Medical Research Center for Phthisiopulmonology and Infection Diseases, 22 Parts’ezda Street, 50, Ekaterinburg 620039, Russia; vakhrusheva2023@yandex.ru (D.V.V.); krasnoborova@urniif.ru (S.Y.K.); dima.dianov.99@mail.ru (D.V.D.); 4 Department of Chemistry, Perm State University, Bukireva Street, 15, Perm 614990, Russia; caterina.stepanova@yandex.ru

**Keywords:** indolo[2,3-*b*]quinoxalines, C–H functionalization, Buchwald–Hartwig amination, antitubercular activity

## Abstract

The synthetic approach based on a sequence of Buchwald–Hartwig cross-coupling and annulation through intramolecular oxidative cyclodehydrogenation has been used for the construction of novel 4-alkyl-4*H*-thieno[2′,3′:4,5]pyrrolo[2,3-*b*]quinoxaline derivatives. For the first time, these polycyclic compounds were evaluated for antimycobacterial activity, including extensively drug-resistant strains. A reasonable bacteriostatic effect against *Mycobacterium tuberculosis* H_37_Rv was demonstrated. A plausible mechanism for antimycobacterial activity of heterocyclic analogues of indolo[2,3-*b*]quinoxalines has been advanced on the basis of their molecular docking data.

## 1. Introduction

**Figure 1:**
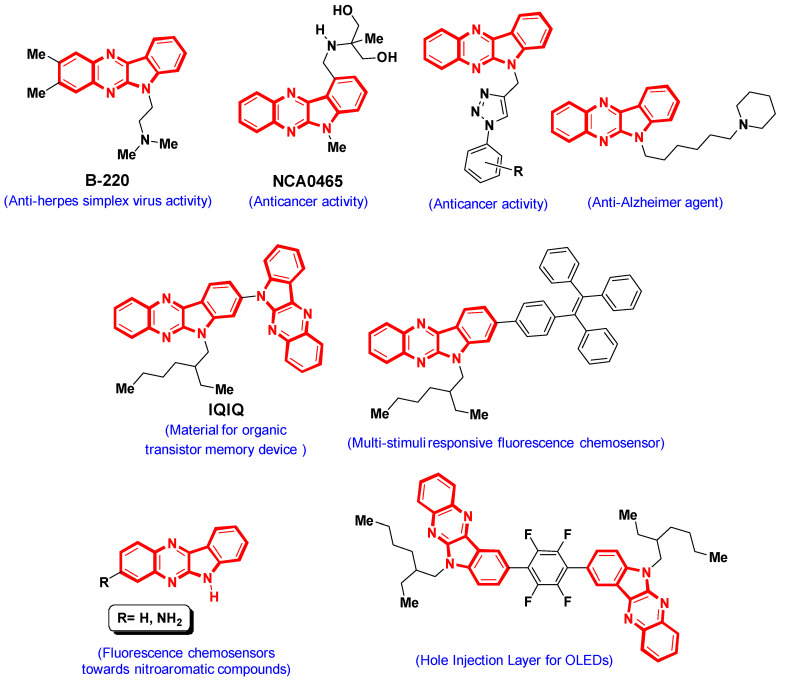
Representative compounds bearing the indoloquinoxaline scaffold.

**Scheme 1:**
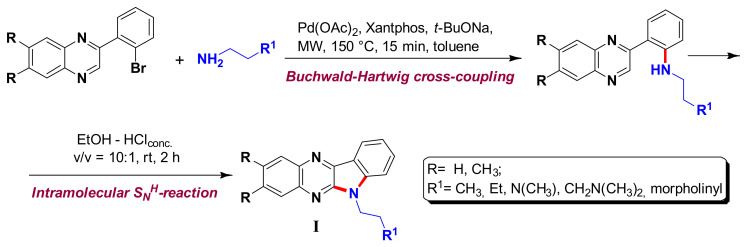
Synthesis of indolo[2,3-*b*]quinoxaline derivatives through the Buchwald–Hartwig cross-coupling and intramolecular S_N_^H^ reactions.

Indolo[2,3-*b*]quinoxaline derivatives are crucial in medicinal chemistry and material science (Figure 1). Compounds of this series have been proven to exhibit antiviral [1,2] (for example, agent B-220), antitumor [3,4,5,6,7,8,9], and antidiabetic [10] properties and can be used for the treatment of neurodegenerative diseases [11]. Additionally, indoloquinoxaline-bearing compounds are used as semiconductors and emitting layers in organic electronics devices [12,13,14,15,16,17,18,19,20,21,22], as well as promising multifunctional chemosensors [23,24]. Several main approaches are based on the annulation of the indole moiety to the quinoxaline core [25,26,27,28]. We have recently [29] proposed a new route for the synthesis of indolo[2,3-*b*]quinoxaline derivatives (**I**) based on the sequential application of the Buchwald–Hartwig cross-coupling and intramolecular nucleophilic aromatic substitution of hydrogen (the so-called S_N_^H^ reaction [30,31]) (Scheme 1). Moderate anticoagulant and tuberculostatic activities have been revealed for the obtained compounds (**I**) [29,32].

**Scheme 2:**
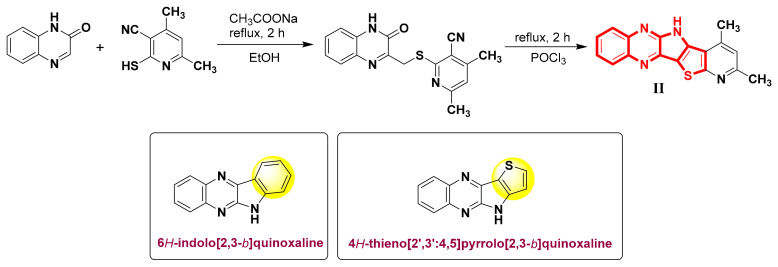
Synthesis of a thieno[2′,3′: 4,5]pyrrolo[2,3-*b*]quinoxaline derivative (**II**) (**on the top**); the structures of 6*H*-indolo[2,3-*b*]quinoxaline and 4*H*-thieno[2′,3′: 4,5]pyrrolo[2,3-*b*]quinoxaline (**on the bottom**).

To the best of our knowledge, the literature describes only one example of the synthesis of compounds bearing the thieno[2′,3′:4,5]pyrrolo[2,3-*b*]quinoxaline scaffold (**II**) (Scheme 2) [33].

In this communication, we wish to report a new synthetic procedure for the preparation of a series of 4-alkyl-4*H*-thieno[2′,3′: 4,5]pyrrolo[2,3-*b*]quinoxalines as new heterocyclic analogues of indolo[2,3-*b*]quinoxalines, and to present the results of their evaluation for antitubercular activity against *Mycobacterium tuberculosis* H_37_Rv.

## 2. Results and Discussion

### 2.1. Synthesis

**Scheme 3:**
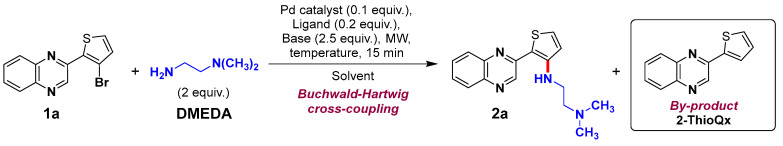
Optimization of conditions for the reaction of **1a** with **DMEDA**.

We have decided to explore an opportunity to use the previously developed two-step approach (see Scheme 1) for the synthesis of the desired 4-alkyl-4*H*-thieno[2′,3′: 4,5]pyrrolo[2,3-*b*]quinoxalines. At the first stage, we have optimized the conditions for the preparation of *N*^1^,*N*^1^-dimethyl-*N*^2^-(2-(quinoxalin-2-yl)thiophen-3-yl)ethane-1,2-diamine (**2a**) by reacting 2-(3-bromo-thiophen-2-yl)quinoxaline (**1a**) with 1,2-dimethylethylenediamine (**DMEDA**) under Buchwald–Hartwig cross-coupling conditions (Scheme 3, Table 1). Using the previously discovered conditions [29] for the synthesis of *N*^1^,*N*^1^-dimethyl-*N*^2^-(2-(quinoxalin-2-yl)phenyl)ethane-1,2-diamine, Pd(OAc)_2_ was used as a catalyst in the presence of tricyclohexyl-phosphine, acting as the ligand, and *t*-BuONa, acting as the base, under microwave synthesis conditions for 15 min (entry 1, Table 1).

The variation of the phosphine ligand has shown that the highest yield of product **2a** is observed in the reaction mixture using 1,1′-ferrocenediyl-*bis*(diphenylphosphine) (Table 1, entries 2–12). By further varying the base, solvent, and temperature, the optimal reaction conditions have been selected for the synthesis of quinoxaline **2a** (Table 1, entries 13–21). It should be noted that, as with the reaction involving 2-(2-bromophenyl)quinoxaline [20], the main issue was related to the side debromination of the starting material **1a**, resulting in the formation of 2-(thiophen-2-yl)quinoxaline (**2-ThioQx**).

**Scheme 4:**
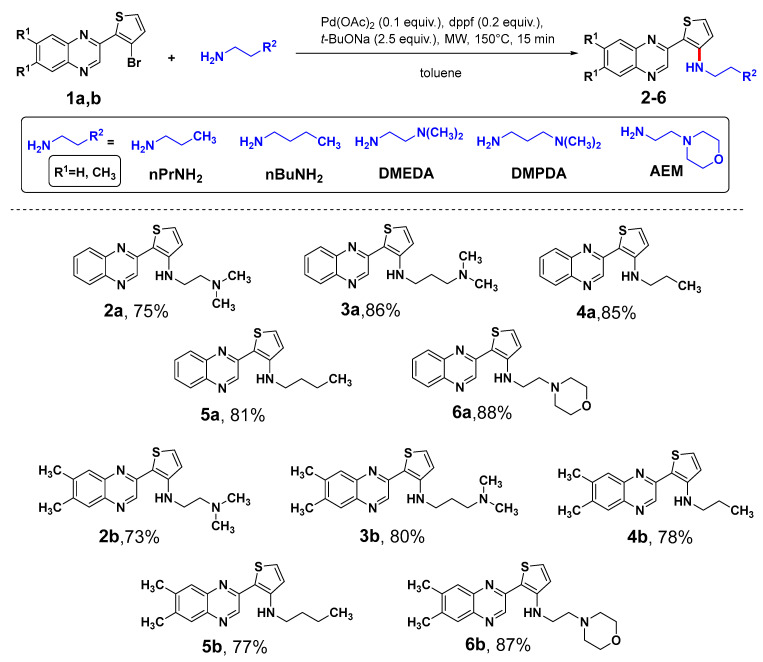
Syntheses of *N*-alkyl-2-(quinoxalin-2-yl)thiophen-3-amines (**2a**–**6a**) and *N*-alkyl-2-(6,7-dimethylquinoxalin-2-yl)thiophen-3-amines (**2b**–**6b**).

Thus, the optimal conditions for the preparation of *N*^1^,*N*^1^-dimethyl-*N*^2^-(2-(quinoxalin-2-yl)thiophen-3-yl)ethane-1,2-diamine (**2a**) in 75% yield proved to be the following: the reaction is carried out in the presence of 0.1 equiv. Pd(OAc)_2_ and 0.2 equiv. dppf in toluene under microwave activation for 15 min (Table 1, entry 20). This procedure has been applied to obtain products with a series of amines, such as 1-propylamine (**nPrNH_2_**), 1-butylamine (**nBuNH_2_**), *N*,*N*-dimethylethylenediamine, *N*,*N*-dimethyl-1,3-propanediamine (**DMPDA**), and 4-(2-aminoethyl)-morpholine (**AEM**). The corresponding *N*-alkyl-2-(quinoxaline-2-yl)anilines (**2a**–**6a**) and *N*-alkyl-2-(6,7-dimethylquinoxaline-2-yl)anilines (**2b**–**6b**) were obtained in high yields (Scheme 4).

The resulting *N*-alkyl-2-(quinoxalin-2-yl)thiophen-3-amines (**2a**–**6a**) and *N*-alkyl-2-(6,7-dimethylquinoxalin-2-yl)thiophen-3-amines (**2b**–**6b**) were formed in situ and subsequently underwent an intramolecular reaction of nucleophilic aromatic substitution of hydrogen (S_N_^H^). This reaction was carried out by treating the reaction mixture with a solution of concentrated hydrochloric acid in ethanol, using a volume ratio of 1:10, in the presence of air as an oxidizing agent, as previously suggested [29].

**Scheme 5:**
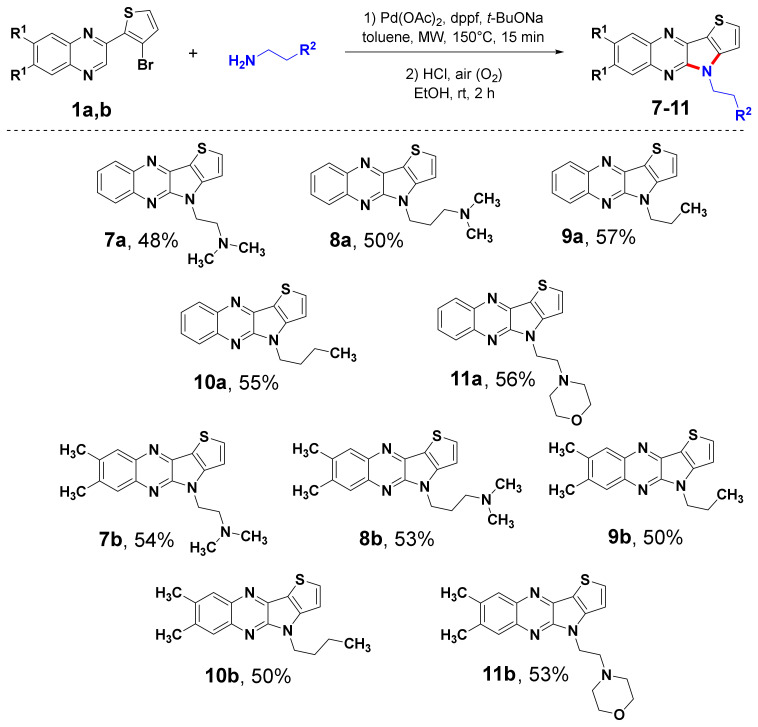
Syntheses of 4-alkyl-4*H*-thieno[2′,3′:4,5]pyrrolo[2,3-*b*]quinoxalines **7**–**11**.

This *one-pot* synthesis allowed us to obtain the target polycyclic indolo[2,3-*b*]quinoxaline analogues, namely 4-alkyl-4*H*-thieno[2′,3′:4,5]pyrrolo[2,3-*b*]quinoxalines **7**–**11**, in good yields (Scheme 5).

### 2.2. Antimycobacterial Activity

The series of compounds **7a**–**11a** and **7b**–**11b** were explored for their activities in vitro against *M. tuberculosis* H_37_Rv (Table 2) with the employment of the aerobic resazurin microplate assay (REMA) [34,35]. The experimental data indicate that most of the tested compounds exhibited relatively low activity, with minimal inhibitory concentrations (MICs) greater than 25 μg/mL. The only 4*H*-thieno[2′,3′:4,5]pyrrolo[2,3-*b*]quinoxaline derivative **7b** that bears the *N*,*N*-dimethylethan-1-amine moiety demonstrated an average value of antitubercular activity of 12.5 μg/mL (Table 2, entry 6). It should be noted that all 4*H*-thieno[2′,3′: 4,5]pyrrolo[2,3-*b*]-quinoxalines **7**–**11** proved to have 2-fold lower values of activities than those reported earlier for similar indolo[2,3-*b*]quinoxaline derivatives [29].

The antitubercular activity of the most active compound **7b** was assessed against the strain *Mycobacterium tuberculosis 5521*, which has extensive drug resistance (XDR) to drugs such as bedaquiline, isoniazid, rifampicin, fluoroquinolones, and others [36]. The minimum inhibitory concentration for compound **7b** against the XDR strain of *Mycobacterium tuberculosis* was found to be 12.5 μg/mL. This indicates the potential for discovering new active agents targeting *Mycobacterium tuberculosis*, particularly those with extensive drug resistance, using such polycyclic derivatives.

An in vitro cytotoxicity test (IC_50_) for the most active agent **7b** was fulfilled on the VERO epithelial cells (ATCC CCL81) using the colorimetric MTT assay (Table 2, Entry 6) [37].

Polycyclic compound **7b** demonstrated a cytotoxicity level that proved to be nearly two times lower than that of the corresponding indolo[2,3-*b*]quinoxaline derivative (IC_50_ = 5.08 ± 1.57 μg/mL) [29] and had a good correlation with the known antitumor activity of similar compounds [3,4,5].

### 2.3. Molecular Docking of Antitubercular Activity

The reverse docking strategy has been used to suggest the mechanism of antimycobacterial activity [29,38]. The complete library of potential targets for *Mycobacterium tuberculosis* H_37_Rv found in the literature has been utilized to realize this idea (Table 3) [39].

Ligands **7a,b**, **8a,b**, and **11a,b** bearing the *N*,*N*-alkylamino moiety were subjected to docking experiments both in non-protonated and protonated forms. Protonated forms are indicated by adding «H» to the number of the corresponding compound (for example, ligand **7aH** means the protonated ligand **7a**). Calculated docking scores are shown in Figure 1 (for each target, docking experiments were performed in triplicate, and each cell corresponds to a mean value).

**Figure 2:**
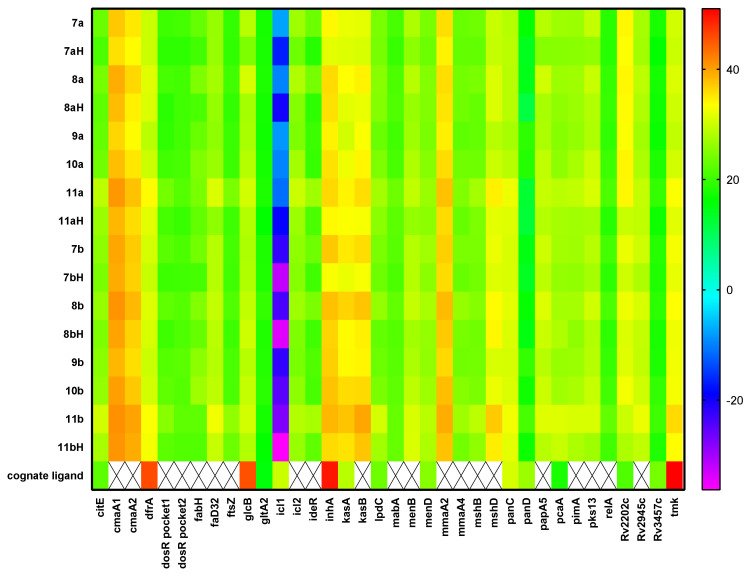
Data of docking scores.

Docking of compounds **7**–**11** to isocitrate lyase (*icl1*) (Table 1, entry 11) demonstrates negative scores (Figure 2), which imply that ligands have a much larger size than the cavity of the binding site. Thus, this protein has been excluded from further consideration.

**Figure 3:**
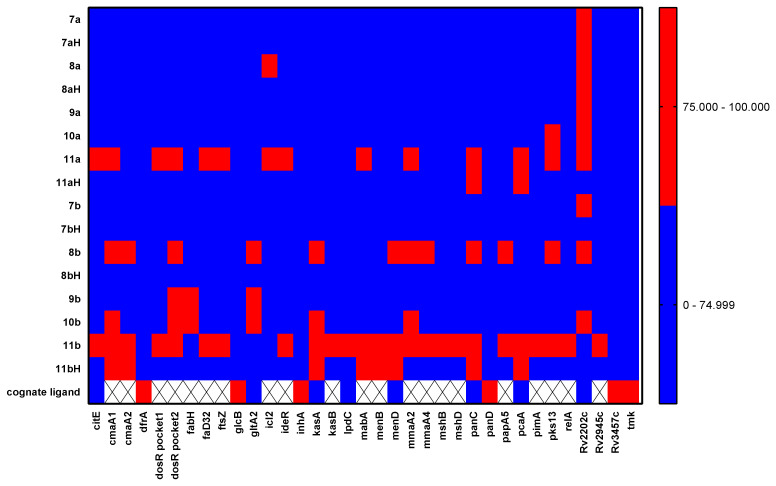
Categorized normalized docking scores.

While executing the reverse docking procedure, we aimed to identify a target that would yield optimal docking scores for the ligands. The docking scores we obtained were normalized and divided into two categories to illustrate the ranking of ligands in relation to each protein (see Figure 3).

**Figure 4:**
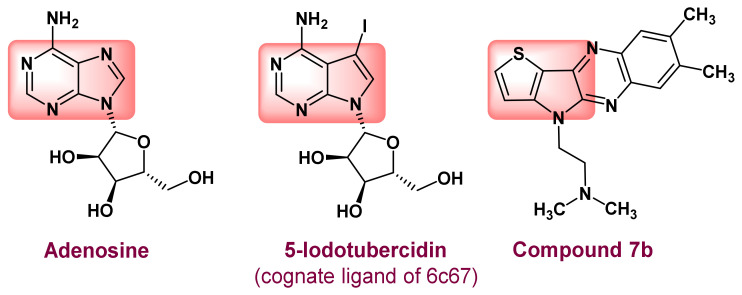
Adenosine and similar structures.

According to Figure 3, compounds possessing the best minimal inhibitory concentration values demonstrate the highest normalized docking scores (top 25%) relative to adenosine kinase (*Rv2202c*) (Table 3, Entry 32). Based on this observation, we have supposed that the estimated target of these substances in *Mycobacterium tuberculosis* appears to be *Rv2202c*. This enzyme generally promotes the transformation of adenosine into adenosine monophosphate in the purine salvage pathway within mycobacteria [40]. The substrate for *Rv2202c* is adenosine, and some adenosine-like substances [41] (Figure 4) can inhibit this enzyme. The considered derivatives **7**–**11** contain an adenosine-like segment (Figure 4), which likely determines good docking scores to *Rv2202c*.

Docking data of derivatives **7**–**11** to the cavity of the enzyme are given in Table 4 (only non-protonated forms are given since the protonated compounds proved to have lower scores, see Figure 2). For comparison, values for 5-iodotubercidin (cognate ligand of *6c67*, see Figure 2) are calculated.

**Figure 5:**
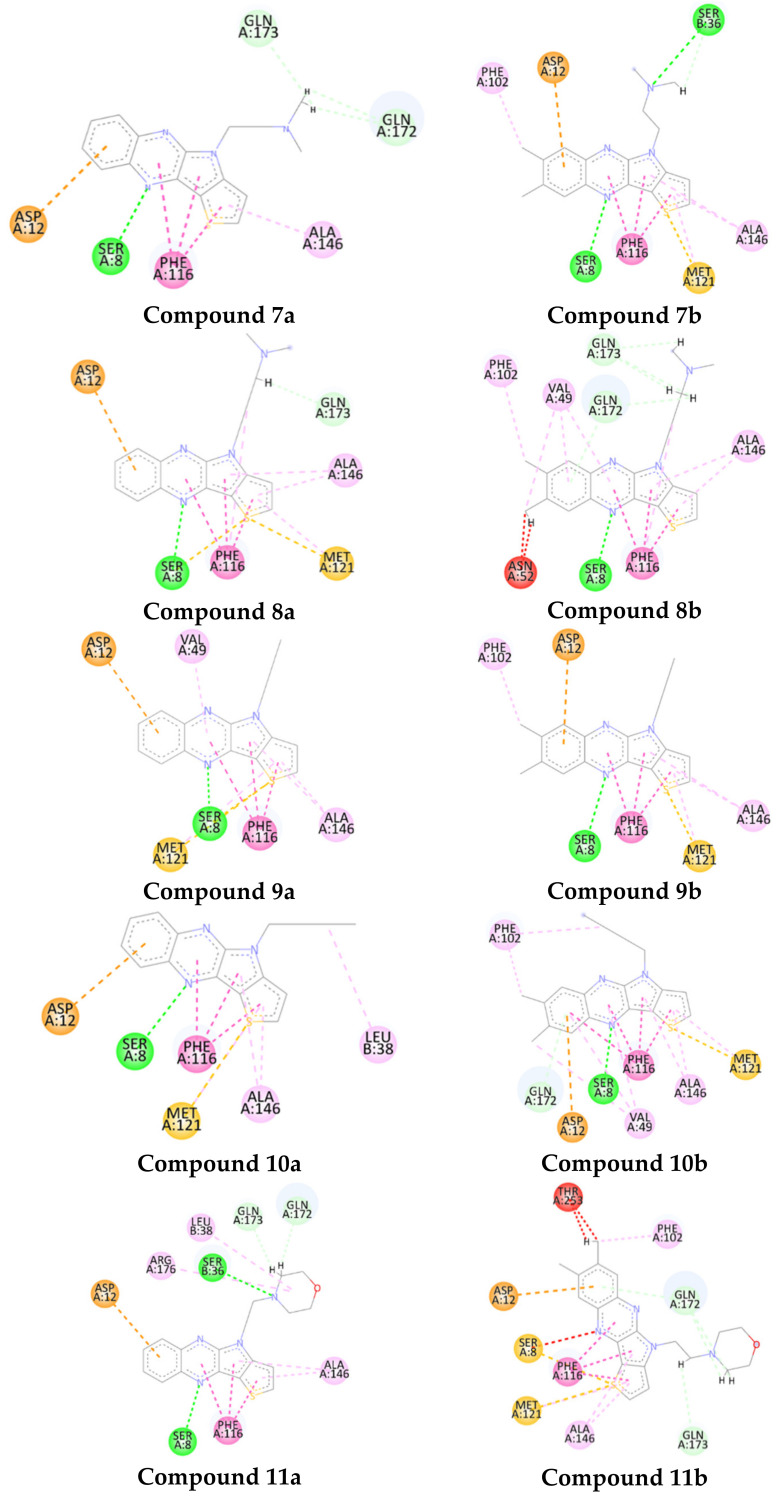
Protein–ligand interactions of compounds **7**–**11** with *Rv2202c* (6c67) (*red*—unfavorable bumps or unfavorable donor–donor interactions, *green*—conventional hydrogen bonds, *pale green*—carbon hydrogen bonds or π–donor hydrogen bonds, *pale pink*—alkyl and π–alkyl interactions, *pink*—π–π stacks, *orange*—π–anion interactions, *yellow*—sulfur–X interactions).

π-stacking interactions display a significant contribution to the binding ability of inhibitors of *Rv2202c* for the enzyme with Phe102 and Phe116 residues [38]. Several hydrogen bonds also contribute to the binding, with residues Gln172, Gln173, Ser8, Ser36′, Asp12, Gly48, Asn52, and Asp257 [38]. In the best-docked poses of compounds **7**–**11**, some of these significant interactions were observed along with some other types of interactions (Figure 5). The best-docked pose of substances **8b** and **11b** shows unfavorable bumps of methyl substituents with Asn52 and Thr253. We have supposed that these unfavorable interactions can be corrected by the flexibility of these amino acid residues. Notably, compounds **7**–**10** demonstrate a hydrogen bond with the binding site (Ser8). Moreover, derivatives **8a**–**10a**, **7b**, **9b**, and **10b** proved to form a sulfur–X bond with the binding site (Met121) due to the presence of a thiophene fragment. Compound **11b** has two sulfur–X bonds with the binding site (Met121, Ser8). This observation aligns well with the results obtained for antimycobacterial activity.

## 3. Materials and Methods

### 3.1. Synthesis

Since we have already described the general data on the analytical equipment and methods used [29], they are presented in the “**General Information**” Section (see Supporting Information). Starting compound **1a** was prepared according to the earlier reported procedure [42]. Details on the antimycobacterial assay, experiments for molecular docking, and the binding sites of the investigated proteins are provided in Supporting Information.

#### 3.1.1. Synthesis of 2-(3-Bromothiophen-2-yl)-6,7-dimethylquinoxaline (**1b**)

A 10 mL microwave reactor vial equipped with a magnetic stirrer bar was charged with selenium dioxide (0.89 g, 8 mmol), 1,4-dioxane/H_2_O 15:1 (16 mL), and 2′-bromoacetophenone (1.64 g, 8 mmol). The vial was capped and placed in the microwave reactor for 1.5 h at 150 °C. After the full conversion of the starting material (checked by TLC) (if needed, an extra 0.1 equiv. of SeO_2_ can be added and heated again for 30 min), the reaction mixture was filtered and the filtrate was concentrated under reduced pressure to give corresponding aryl glyoxal as a dark orange oil. The residue was transferred with EtOH (10 mL) in a round-bottom flask charged with a magnetic stirrer and 4,5-dimethyl-1,2-phenylenediamin (0.76 g, 5.6 mmol) was added. The mixture was refluxed for 1 h. After that, the mixture was cooled down, and the precipitate was filtered off, washed with ethanol, and then air-dried. Yield: 67% (1205 g). Physical State: beige solid. ^1^H NMR (500 MHz, CDCl_3_): δ 9.67 (s, 1H), 7.85 (d, *J* = 7.1 Hz, 2H), 7.46 (d, *J* = 5.3 Hz, 1H), 7.14 (d, *J* = 5.3 Hz, 1H), 2.51 (s, 6H). ^13^C NMR (125 MHz, CDCl_3_): δ 149.9, 142.2, 141.1, 141.1, 140.7, 140.4, 137.1, 132.8, 128.8, 128.3, 128.2, 109.2, 20.4, 20.3. MP: 135–136 °C. HRMS (ESI): calc’d for C_14_H_12_BrN_2_S [M+H]^+^ *m*/*z* 318.9899, found 318.9899. *R*_f_ = 0.44 (10% EtOAc/hexanes). Purification: recrystallization from ethanol.

#### 3.1.2. General Procedure for the Synthesis of N-Alkyl-2-(quinoxaline-2-yl)thiophen-3-amines (**2a**–**6a**) and N-Alkyl-2-(6,7-dimethylquinoxalin-2-yl)thiophen-3-amines (**2b**–**6b**)

A 10 mL microwave reactor vial equipped with a magnetic stirrer bar was charged with 50 mg of quinoxaline (1.72 mmol), the corresponding amine (2.0 equiv), Pd(OAc)_2_ (0.1 equiv.), dppf (0.2 equiv.), *t*-BuONa (2.5 equiv.), and 6 mL of toluene sparged with argon for 10 min. The empty volume of the vial was also spared with argon, the cap was quickly sealed, and then the vial was placed in a microwave reactor for 15 min. After that, the reaction mixture was filtered through a pad of Celite, washed with CH_2_Cl_2_, and concentrated under reduced pressure. Purification of the residue by flash column chromatography (silica gel) provided the products **2a**–**6a** and **2b**–**6b**.

#### 3.1.3. N^1^,N^1^-Dimethyl-N^2^-(2-(quinoxalin-2-yl)thiophen-3-yl)ethane-1,2-diamine (**2a**)

Yield: 75% (38 mg). Physical State: orange solid. ^1^H NMR (500 MHz, CDCl_3_): δ 8.97–8.92 (m, 1H), 8.87 (s, 1H), 7.95 (dd, *J* = 8.3, 1.1 Hz, 1H), 7.87 (dd, *J* = 8.4, 1.1 Hz, 1H), 7.66 (ddd, *J* = 8.3, 7.0, 1.4 Hz, 1H), 7.54 (ddd, *J* = 8.2, 7.0, 1.3 Hz, 1H), 7.36 (d, *J* = 5.5 Hz, 1H), 6.81 (d, *J* = 5.4 Hz, 1H), 3.49 (q, *J* = 6.1 Hz, 2H), 2.68 (t, *J* = 6.3 Hz, 2H), 2.34 (s, 6H). ^13^C NMR (125 MHz, CDCl_3_): δ 153.0, 150.2, 144.0, 141.2, 139.1, 130.0, 129.0, 128.3, 127.6, 127.0, 117.5, 105.7, 58.7, 45.5, 43.3. MP: 103–104 °C. HRMS (ESI): calc’d for C_16_H_19_N_4_S [M+H]^+^ *m*/*z* 299.1325, found 299.1323. *R*_f_ = 0.26 (50% EtOAc/hexanes + 1% TEA). Purification: (SiO_2_, 50% EtOAc/hexanes + 1% TEA).

#### 3.1.4. N^1^,N^1^-Dimethyl-N^3^-(2-(quinoxalin-2-yl)thiophen-3-yl)propane-1,3-diamine (**3a**)

Yield: 86% (46 mg). Physical State: orange semisolid. ^1^H NMR (500 MHz, CDCl_3_): δ 8.9 (s, 1H), 8.73–8.67 (m, 1H), 7.96 (dd, *J* = 8.2, 1.0 Hz, 1H), 7.87 (dd, *J* = 8.3, 0.8 Hz, 1H), 7.66 (ddd, *J* = 8.3, 7.0, 1.3 Hz, 1H), 7.54 (ddd, *J* = 8.2, 7.0, 1.3 Hz, 1H), 7.36 (d, *J* = 5.4 Hz, 1H), 6.84 (d, *J* = 5.5 Hz, 1H), 3.50 (q, *J* = 6.4 Hz, 2H), 2.57 (t, *J* = 7.3 Hz, 2H), 2.35 (s, 6H), 1.97 (quint, *J* = 7.1 Hz, 2H). ^13^C NMR (125 MHz, CDCl_3_): δ 153.0, 150.2, 144.1, 141.0, 139.1, 130.0, 129.1, 128.5, 127.4, 127.1, 117.4, 105.7, 57.0, 45.2, 43.3, 27.8. MP: -. HRMS (ESI): calc’d for C_17_H_21_N_4_S [M+H]^+^ *m*/*z* 313.1481, found 313.1481. *R*_f_ = 0.10 (50% EtOAc/hexanes + 1% TEA). Purification: (SiO_2_, 50% EtOAc/hexanes + 1% TEA).

#### 3.1.5. N-Propyl-2-(quinoxalin-2-yl)thiophen-3-amine (**4a**)

Yield: 85% (39 mg). Physical State: orange solid. ^1^H NMR (500 MHz, CDCl_3_): δ 8.87 (s, 1H), 8.76–8.69 (m, 1H), 7.95 (dd, *J* = 8.2, 1.3 Hz, 1H), 7.83 (dd, *J* = 8.3, 1.0 Hz, 1H), 7.65 (ddd, *J* = 8.3, 7.0, 1.4 Hz, 1H), 7.53 (ddd, *J* = 8.2, 6.9, 1.3 Hz, 1H), 7.35 (d, *J* = 5.5 Hz, 1H), 6.81 (d, *J* = 5.6 Hz, 1H), 3.38 (dt, *J* = 5.7, 6.8 Hz, 2H), 1.79 (sext, *J* = 7.2 Hz, 2H), 1.1 (t, *J* = 7.4 Hz, 3H). ^13^C NMR (125 MHz, CDCl_3_): δ 153.3, 150.3, 144.2, 141.1, 139.1, 130.0, 129.1, 128.4, 127.4, 127.0, 117.4, 105.3, 47.2, 23.3, 11.7. MP: 79–80 °C. HRMS (ESI): calc’d for C_15_H_16_N_3_S [M+H]^+^ *m*/*z* 270.1059, found 270.1058. *R*_f_ = 0.24 (5% EtOAc/hexanes). Purification: (SiO_2_, 5% EtOAc/hexanes).

#### 3.1.6. N-Butyl-2-(quinoxalin-2-yl)thiophen-3-amine (**5a**)

Yield: 81% (39 mg). Physical State: orange solid. ^1^H NMR (500 MHz, CDCl_3_): δ 8.87 (s, 1H), 8.73–8.67 (m, 1H), 7.95 (dd, *J* = 8.2, 1.1 Hz, 1H), 7.82 (dd, *J* = 8.3, 1.1 Hz, 1H), 7.65 (ddd, *J* = 8.3, 7.0, 1.3 Hz, 1H), 7.74 (ddd, *J* = 8.2, 7.0, 1.3 Hz, 1H), 7.35 (d, *J* = 5.5 Hz, 1H), 6.81 (d, *J* = 5.6 Hz, 1H), 3.41 (dt, *J* = 5.7, 6.8 Hz, 2H), 1.75 (quint, *J* = 7.2 Hz, 2H), 1.55 (sext, *J* = 7.4 Hz, 2H), 1.02 (t, *J* = 7.4 Hz, 3H). ^13^C NMR (125 MHz, CDCl_3_): δ 153.4, 150.3, 144.2, 141.1, 139.1, 130.0, 129.1, 128.4, 127.4, 127.0, 117.4, 105.3, 45.0, 32.1, 20.2, 13.9. MP: 83–84 °C. HRMS (ESI): calc’d for C_16_H_18_N_3_S [M+H]^+^ *m*/*z* 284.1216, found 284.1215. *R*_f_ = 0.27 (5% EtOAc/hexanes). Purification: (SiO_2_, 5% EtOAc/hexanes).

#### 3.1.7. N-(2-Morpholinoethyl)-2-(quinoxalin-2-yl)thiophen-3-amine (**6a**)

Yield: 88% (51 mg). Physical State: orange solid. ^1^H NMR (500 MHz, CDCl_3_): δ 8.89 (s, 1H), 8.85–8.80 (m, 1H), 7.97 (dd, *J* = 8.5, 1.1 Hz, 1H), 7.95 (dd, *J* = 8.4, 1.1 Hz, 1H) 7.67 (ddd, *J* = 8.3, 7.0, 1.3 Hz, 1H), 7.56 (ddd, *J* = 8.3, 6,9, 1.3 Hz, 1H), 7.37 (d, *J* = 5.4 Hz, 1H), 6.82 (d, *J* = 5.3 Hz, 1H), 3.79 (t, *J* = 4.6 Hz, 4H), 3.52 (q, *J* = 5.85 Hz, 2H), 2.75 (t, *J* = 6.1 Hz, 2H), 2.59 (br s, 4H). ^13^C NMR (125 MHz, CDCl_3_): δ 152.7, 150.2, 144.1, 141.2, 139.1, 130.0, 129.1, 128.4, 127.5, 127.1, 117.6, 106.0, 67.1, 57.8, 53.6, 42.2. MP: 130–131 °C. HRMS (ESI): calc’d for C_18_H_21_N_4_OS [M+H]^+^ *m*/*z* 341.1431, found 341.1430. *R*_f_ = 0.37 (% EtOAc/hexanes). Purification: (SiO_2_, 50% EtOAc/hexanes).

#### 3.1.8. N^1^-(2-(6,7-Dimethylquinoxalin-2-yl)thiophen-3-yl)-N^2^,N^2^-dimethylethane-1,2-diamine (**2b**)

Yield: 73% (36 mg). Physical State: orange-brown solid. ^1^H NMR (500 MHz, CDCl_3_): δ 8.90–8.83 (m, 1H), 8.78 (s, 1H), 7.69 (s, 1H), 7.63 (s, 1H), 7.31 (d, *J* = 5.4 Hz, 1H), 6.80 (d, *J* = 5.4 Hz, 1H), 3.49 (q, *J* = 6.0 Hz, 2H), 2.69 (t, *J* = 6.4 Hz, 2H), 2.46 (s, 3H), 2.44 (s, 3H), 2.40 (s, 6H). ^13^C NMR (125 MHz, CDCl_3_): δ 152.3, 149.5, 142.9, 140.1, 139.9, 138.0, 137.2, 128.3, 127.5, 127.1, 117.5, 106.0, 58.7, 45.5, 43.3, 20.4, 20.1. MP: 82–83 °C. HRMS (ESI): calc’d for C_18_H_23_N_4_S [M+H]^+^ *m*/*z* 327.1638, found 327.1638. *R*_f_ = 0.21 (50% EtOAc/hexanes + 1% TEA). Purification: (SiO_2_, 50% EtOAc/hexanes).

#### 3.1.9. N^1^-(2-(6,7-Dimethylquinoxalin-2-yl)thiophen-3-yl)-N^3^,N^3^-dimethylpropane-1,3-diamine (**3b**)

Yield: 80% (41 mg). Physical State: orange solid. ^1^H NMR (500 MHz, CDCl_3_): δ 8.79 (s, 1H), 8.66–8.58 (m, 1H), 7.70 (s, 1H), 7.62 (s, 1H), 7.31 (d, *J* = 5.4 Hz, 1H), 6.82 (d, *J* = 5.4 Hz, 1H), 3.46 (q, *J* = 6.3 Hz, 2H), 2.48 (t, *J* = 7.0 Hz, 2H), 2.46 (s, 3H), 2.44 (s, 3H), 2.29 (s, 6H), 1.92 (quint, *J* = 7.0 Hz, 2H). ^13^C NMR (125 MHz, CDCl_3_): δ 152.6, 149.6, 143.1, 140.2, 139.8, 138.0, 137.2, 128.4, 127.6, 126.9, 117.5, 105.9, 57.3, 45.6, 43.5, 28.3, 20.2, 20.0. MP: 76–77 °C. HRMS (ESI): calc’d for C_19_H_25_N_4_S [M+H]^+^ *m*/*z* 341.1794, found 341.1794. *R*_f_ = 0.12 (50% EtOAc/hexanes + 1% TEA). Purification: (SiO_2_, 50% EtOAc/hexanes).

#### 3.1.10. 2-(6,7-Dimethylquinoxalin-2-yl)-N-propylthiophen-3-amine (**4b**)

Yield: 78% (35 mg). Physical State: yellow-orange solid. ^1^H NMR (500 MHz, CDCl_3_): δ 8.78 (s, 1H), 8.72–8.54 (m, 1H), 7.69 (s, 1H), 7.58 (s, 1H), 7.31 (d, *J* = 5.4 Hz, 1H), 6.80 (d, *J* = 5.4 Hz, 1H), 3.37 (t, *J* = 7.0 Hz, 2H), 2.45 (s, 3H), 2.44 (s, 3H), 1.78 (sext, *J* = 7.2 Hz, 2H), 1.10 (t, *J* = 7.5 Hz, 3H). ^13^C NMR (125 MHz, CDCl_3_): δ 152.7, 149.6, 143.1, 140.2, 139.8, 138.0, 137.2, 128.4, 127.6, 126.8, 117.5, 105.6, 47.2, 23.4, 20.3, 20.0, 11.7. MP: 118–119 °C. HRMS (ESI): calc’d for C_17_H_20_N_3_S [M+H]^+^ *m*/*z* 298.1372, found 298.1373. *R*_f_ = 0.20 (% EtOAc/hexanes). Purification: (SiO_2_, 40% → 50% EtOAc/hexanes).

#### 3.1.11. N-Butyl-2-(6,7-dimethylquinoxalin-2-yl)thiophen-3-amine (**5b**)

Yield: 77% (36 mg). Physical State: orange solid. ^1^H NMR (500 MHz, CDCl_3_): δ 8.78 (s, 1H), 8.71–8.49 (m, 1H), 7.69 (s, 1H), 7.58 (s, 1H), 7.31 (d, *J* = 5.5 Hz, 1H), 6.80 (d, *J* = 5.6 Hz, 1H), 3.40 (t, *J* = 7.0 Hz, 2H), 2.46 (s, 3H), 2.44 (s, 3H), 1.75 (quint, *J* = 7.2 Hz, 2H), 1.54 (sext, *J* = 7.4 Hz, 2H), 1.02 (t, *J* = 7.5 Hz, 3H). ^13^C NMR (125 MHz, CDCl_3_): δ 152.7, 149.6, 143.1, 140.2, 139.8, 138.0, 137.2, 128.4, 127.6, 126.8, 117.4, 105.6, 45.1, 32.2, 20.3, 20.3, 20.0, 13.7. MP: 80–81 °C. HRMS (ESI): calc’d for C_18_H_22_N_3_S [M+H]^+^ *m*/*z* 312.1529, found 312.1527. *R*_f_ = 0.21 (% EtOAc/hexanes). Purification: (SiO_2_, 40% → 50% EtOAc/hexanes).

#### 3.1.12. 2-(6,7-Dimethylquinoxalin-2-yl)-N-(2-morpholinoethyl)thiophen-3-amine (**6b**)

Yield: 87% (48 mg). Physical State: orange solid. ^1^H NMR (500 MHz, CDCl_3_): δ 8.80 (s, 1H), 8.78–8.74 (m, 1H), 7.72 (d, *J* = 7.7 Hz, 2H), 7.32 (d, *J* = 5.4 Hz, 1H), 6.80 (d, *J* = 5.4 Hz, 1H), 3.81 (t, *J* = 4.6 Hz, 4H), 3.50 (q, *J* = 5.9 Hz, 2H), 2.75 (t, *J* = 6.1 Hz, 2H), 2.62–2.56 (m, 4H), 2.47 (s, 3H), 2.45 (s, 3H). ^13^C NMR (125 MHz, CDCl_3_): δ 152.1, 149.5, 143.0, 140.2, 139.9, 138.1, 137.3, 128.4, 127.5, 127.0, 117.6, 106.3, 67.1, 57.9, 53.6, 42.2, 20.3, 20.0. MP: 149–150 °C. HRMS (ESI): calc’d for C_20_H_25_N_4_OS [M+H]^+^ *m*/*z* 369.1744, found 369.1742. *R*_f_ = 0.26 (50% EtOAc/hexanes). Purification: (SiO_2_, 50% EtOAc/hexanes).

#### 3.1.13. General Procedure for Syntheses of 4-Alkyl-4H-thieno[2′,3′:4,5]pyrrolo[2,3-b]quinoxaline Derivatives (**7**–**11**)

A 10 mL microwave reactor vial equipped with a magnetic stirrer bar was charged with 50 mg of quinoxaline (3.34 mmol), the corresponding amine (2.0 equiv), Pd(OAc)_2_ (10 mol.%), dppf (20 mol.%), *t*-BuONa (2.5 equiv.), and 6 mL of toluene sparged with argon for 40 min. The empty volume of the vials was also spared with argon, the cap was quickly sealed, and then the vial was placed in a microwave reactor for 15 min. The reaction was carried out twice and the reaction mixtures were combined. After, the reaction mixture was filtered through a pad of Celite, washed with CH_2_Cl_2_, and concentrated under reduced pressure. The crude product was dissolved in 10 mL of ethanol with 1 mL of concentrated hydrochloric acid and stirred at 80 °C for a few days. After reaction completion (monitored by TLC), NaHCO_3_ was added to the reaction mixture until the end of the gas evolution. The mixture was filtered through a cotton plug, washed with CH_2_Cl_2_, and concentrated under reduced pressure. Purification of the residue by flash column chromatography (silica gel) provided the products **7a**–**11a** and **7b**–**11b**.

#### 3.1.14. N,N-Dimethyl-2-(4H-thieno[2′,3′:4,5]pyrrolo[2,3-b]quinoxalin-4-yl)ethan-1-amine (**7a**)

Yield: 48% (49 mg). Physical State: pale orange solid. ^1^H NMR (500 MHz, CDCl_3_): δ 8.22 (dd, *J* = 8.2, 1.7 Hz, 1H), 8.11 (dd, *J* = 8.0, 1.6 Hz, 1H), 7.79 (d, *J* = 5.2 Hz, 1H), 7.72–7.64 (m, 2H), 7.25 (d, *J* = 5.2 Hz, 1H), 4.60 (t, *J* = 6.73 Hz, 2H), 2.86 (t, *J* = 7.0 Hz, 2H), 2.36 (s, 6H). ^13^C NMR (125 MHz, CDCl_3_): δ 152.3, 146.7, 139.2, 138.7, 137.4, 134.2, 128.8, 128.0, 127.6, 126.3, 112.6, 111.4 58.0, 45.7, 41.8. MP: 95–96 °C. HRMS (ESI): calc’d for C_16_H_17_N_4_S [M+H]^+^ *m*/*z* 297.1168, found 297.1167. *R*_f_ = 0.13 (50% EtOAc/hexanes + 1% TEA). Purification: (SiO_2_, 40% → 50% EtOAc/hexanes).

#### 3.1.15. N,N-Dimethyl-3-(4H-thieno[2′,3′:4,5]pyrrolo[2,3-b]quinoxalin-4-yl)propan-1-amine (**8a**)

Yield: 50% (53 mg). Physical State: green-orange solid. ^1^H NMR (500 MHz, CDCl_3_): δ 8.22 (dd, *J* = 8.1, 1.7 Hz, 1H), 8.12 (dd, *J* = 8.0, 1.7 Hz, 1H), 7.80 (d, *J* = 5.2 Hz, 1H), 7.71–7.65 (m, 2H), 7.28 (d, *J* = 5.2 Hz, 1H), 4.56 (t, *J* = 6.9 Hz, 2H), 2.34 (t, *J* = 6.7 Hz, 2H), 2.23 (s, 6H), 2.14 (quint, *J* = 6.9 Hz, 2H). ^13^C NMR (125 MHz, CDCl_3_): δ 152.6, 146.7, 139.1, 138.7, 137.4, 134.1, 128.8, 127.9, 127.5, 126.3, 112.2, 111.5, 56.5, 45.4, 41.5, 27.3. MP: 78–79 °C. HRMS (ESI): calc’d for C_17_H_19_N_4_S [M+H]^+^ *m*/*z* 311.1325, found 311.1324. *R*_f_ = 0.07 (50% EtOAc/hexanes + 1% TEA). Purification: (SiO_2_, 50% EtOAc/hexanes).

#### 3.1.16. 4-Propyl-4H-thieno[2′,3′:4,5]pyrrolo[2,3-b]quinoxaline (**9a**)

Yield: 57% (52 mg). Physical State: orange-yellow solid. ^1^H NMR (500 MHz, CDCl_3_): δ 8.22 (d, *J* = 8.2, 1.7 Hz, 1H), 8.12 (dd, *J* = 7.9, 1.7 Hz, 1H), 7.80 (d, *J* = 5.1 Hz, 1H), 7.72–7.64 (m, 2H), 7.22 (d, *J* = 5.2 Hz, 1H), 4.46 (t, *J* = 7.2 Hz, 2H), 2.00 (sext, *J* = 7.3 Hz, 2H), 1.00 (t, *J* = 7.3 Hz, 3H). ^13^C NMR (125 MHz, CDCl_3_): δ 152.4, 146.7, 139.1, 138.7, 137.3, 134.2, 128.8, 127.9, 127.5, 126.2, 112.3, 111.3, 45.1, 22.7, 11.5. MP: 140–141 °C. HRMS (ESI): calc’d for C_15_H_14_N_3_S [M+H]^+^ *m*/*z* 268.0903, found 268.0906. *R*_f_ = 0.10 (5% EtOAc/hexanes). Purification: (SiO_2_, 5% EtOAc/hexanes).

#### 3.1.17. 4-Butyl-4H-thieno[2′,3′:4,5]pyrrolo[2,3-b]quinoxaline (**10a**)

Yield: 55% (53 mg). Physical State: yellow-orange solid. ^1^H NMR (500 MHz, CDCl_3_): δ 8.22 (dd, *J* = 7.9, 1.7 Hz, 1H), 8.12 (dd, *J* = 8.0, 1.7 Hz, 1H), 7.80 (d, *J* = 5.2 Hz, 1H), 7.72–7.64 (m, 2H), 7.22 (d, *J* = 5.1 Hz, 1H), 4.50 (t, *J* = 7.0 Hz, 2H), 1.95 (quint, *J* = 7.4 Hz, 2H), 1.41 (sext, *J* = 7.5 Hz, 2H), 0.97 (t, *J* = 7.3, 3H). ^13^C NMR (125 MHz, CDCl_3_): δ 152.3, 146.7, 139.1, 138.7, 137.3, 134.2, 128.8, 128.0, 127.5, 126.2, 112.3, 111.3, 43.3, 31.5, 20.2, 13.7. MP: 77–78 °C. HRMS (ESI): calc’d for C_16_H_16_N_3_S [M+H]^+^ *m*/*z* 282.1059, found 282.1061. *R*_f_ = 0.13 (5% EtOAc/hexanes). Purification: (SiO_2_, 5% EtOAc/hexanes).

#### 3.1.18. 4-(2-(4H-Thieno[2′,3′:4,5]pyrrolo[2,3-b]quinoxalin-4-yl)ethyl)morpholine (**11a**)

Yield: 56% (65 mg). Physical State: pale orange solid. ^1^H NMR (500 MHz, CDCl_3_): δ 8.22 (dd, *J* = 7.8, 1.9 Hz, 1H), 8.09 (dd, *J* = 8.0, 1.7 Hz, 1H), 7.80 (d, *J* = 5.2 Hz, 1H), 7.73–7.65 (m, 2H), 7.24 (d, *J* = 5.2 Hz, 1H), 4.61 (t, *J* = 6.5 Hz, 2H), 3.61 (t, *J* = 4.5 Hz, 4H), 2.88 (t, *J* = 6.6 Hz, 2H), 2.67 (t, *J* = 4.1 Hz, 4H). ^13^C NMR (125 MHz, CDCl_3_): δ 152.3, 146.8, 139.2, 138.6, 137.4, 134.1, 128.8, 127.9, 127.6, 126.3, 112.5, 111.4, 66.9, 57.3, 53.8, 41.0. MP: 148–149 °C. HRMS (ESI): calc’d for C_18_H_19_N_4_OS [M+H]^+^ *m*/*z* 339.1274, found 339.1276. *R*_f_ = 0.15 (% EtOAc/hexanes). Purification: (SiO_2_, 40% → 50% EtOAc/hexanes).

#### 3.1.19. 2-(7,8-Dimethyl-4H-thieno[2′,3′:4,5]pyrrolo[2,3-b]quinoxalin-4-yl)-N,N-dimethylethan-1-amine (**7b**)

Yield: 54% (60 mg). Physical State: green-orange solid. ^1^H NMR (500 MHz, CDCl_3_): δ 7.97 (s, 1H), 7.88 (s, 1H), 7.76 (d, *J* = 5.2 Hz, 1H). 7.25 (d, *J* = 5.2 Hz, 1H), 4.60 (t, *J* = 6.9 Hz, 2H), 2.87 (t, *J* = 7.0 Hz, 2H), 2.53 (s, 3H), 2.53 (s, 3H), 2.37 (s, 6H). ^13^C NMR (125 MHz, CDCl_3_): δ 151.5, 146.4, 138.1, 137.9, 137.4, 136.6, 136.3, 133.3, 127.9, 127.1, 112.6, 111.3, 58.0, 45.7, 41.7, 20.3, 20.2. MP: 141–142 °C. HRMS (ESI): calc’d for C_18_H_21_N_4_S [M+H]^+^ *m*/*z* 325.1481, found 325.1483. *R*_f_ = 0.13 (50% EtOAc/hexanes + 1% TEA). Purification: (SiO_2_, 50% EtOAc/hexanes).

#### 3.1.20. 3-(7,8-Dimethyl-4H-thieno[2′,3′:4,5]pyrrolo[2,3-b]quinoxalin-4-yl)-N,N-dimethylpropan-1-amine (**8b**)

Yield: 53% (62 mg). Physical State: pale yellow solid. ^1^H NMR (500 MHz, CDCl_3_): δ 7.96 (s, 1H), 7.87 (s, 1H), 7.74 (d, *J* = 5.1 Hz, 1H), 7.25 (d, *J* = 5.1 Hz, 1H), 4.53 (t, *J* = 6.9 Hz, 2H), 2.52 (s, 6H), 2.33 (t, *J* = 7.0 Hz, 2H), 2.22 (s, 6H), 2.12 (quint, *J* = 6.9, 2H). ^13^C NMR (125 MHz, CDCl_3_): δ 151.8, 146.4, 138.0, 137.9, 137.4, 136.6, 136.3, 133.2, 127.9, 127.1, 112.2, 111.4, 56.5, 45.4, 41.4, 27.4, 20.3, 20.2. MP: 126–127 °C. HRMS (ESI): calc’d for C_19_H_23_N_4_S [M+H]^+^ *m*/*z* 339.1638, found 339.1639. *R*_f_ = 0.07 (50% EtOAc/hexanes + 1% TEA). Purification: (SiO_2_, 50% EtOAc/hexanes).

#### 3.1.21. 7,8-Dimethyl-4-propyl-4H-thieno[2′,3′:4,5]pyrrolo[2,3-b]quinoxaline (**9b**)

Yield: 50% (51 mg). Physical State: yellow solid. ^1^H NMR (500 MHz, CDCl_3_): δ 7.96 (s, 1H), 7.87 (s, 1H), 7.75 (d, *J* = 5.2 Hz, 1H), 7.19 (d, *J* = 5.2 Hz, 1H), 4.43 (t, *J* = 7.1 Hz, 2H), 2.51 (s, 6H), 1.99 (sext, *J* = 7.3 Hz, 2H), 0.99 (t, *J* = 7.5 Hz, 3H). ^13^C NMR (125 MHz, CDCl_3_): δ 151.1, 146.0, 137.5, 137.4, 137.0, 136.1, 135.8, 132.8, 127.4, 126.6, 111.8, 110.7, 44.6, 22.3, 19.8, 19.7, 11.0. MP: 119–120 °C. HRMS (ESI): calc’d for C_17_H_18_N_3_S [M+H]^+^ *m*/*z* 296.1216, found 296.1215. *R*_f_ = 0.13 (5% EtOAc/hexanes). Purification: (SiO_2_, 40% → 50% EtOAc/hexanes).

#### 3.1.22. 4-Butyl-7,8-dimethyl-4H-thieno[2′,3′:4,5]pyrrolo[2,3-b]quinoxaline (**10b**)

Yield: 50% (53 mg). Physical State: pale orange solid. ^1^H NMR (500 MHz, CDCl_3_): δ 7.96 (s, 1H), 7.87 (s, 1H), 7.75 (d, *J* = 5.1 Hz, 1H), 7.19 (d, *J* = 5.2 Hz, 1H), 4.47 (t, *J* = 7.2 Hz, 2H), 2.51 (s, 6H), 1.97–1.89 (m, 2H), 1.40 (sext, *J* = 7.5 Hz, 2H), 0.96 (t, *J* = 7.3 Hz, 3H). ^13^C NMR (125 MHz, CDCl_3_): δ 151.1, 146.0, 137.5, 137.4, 137.0, 136.1, 135.8, 132.8, 127.4, 126.6, 111.8, 110.7, 42.7, 31.0, 29.2, 19.8, 19.7, 13.3. MP: 142–143 °C. HRMS (ESI): calc’d for C_18_H_20_N_3_S [M+H]^+^ *m*/*z* 310.1372, found 310.1373. *R*_f_ = 0.19 (% EtOAc/hexanes). Purification: (SiO_2_, CH_2_Cl_2_).

#### 3.1.23. 4-(2-(7,8-Dimethyl-4H-thieno[2′,3′:4,5]pyrrolo[2,3-b]quinoxalin-4-yl)ethyl)morpholine (**11b**)

Yield: 53% (67 mg). Physical State: pale yellow solid. ^1^H NMR (500 MHz, CDCl_3_): δ 7.96 (s, 1H), 7.84 (s, 1H), 7.75 (d, *J* = 5.2 Hz, 1H). 7.21 (d, *J* = 5.2 Hz, 1H), 4.58 (t, *J* = 6.6 Hz, 2H), 3.61 (t, *J* = 4.4 Hz, 4H), 2.87 (t, *J* = 6.5 Hz, 2H), 2.59–2.54 (m, 4H), 2.52 (s, 6H). ^13^C NMR (125 MHz, CDCl_3_): δ 151.5, 146.5, 138.1, 138.0, 137.4, 136.6, 136.4, 133.2, 127.9, 127.0, 112.6, 111.3, 66.9, 57.3, 53.8, 40.9, 20.3, 20.2. MP: 166–167 °C. HRMS (ESI): calc’d for C_20_H_23_N_4_OS [M+H]^+^ *m*/*z* 367.1587, found 367.1587. *R*_f_ = 0.17 (50% EtOAc/hexanes). Purification: (SiO_2_, 50% EtOAc/hexanes).

### 3.2. Evaluation of Antimycobacterial Activity and Molecular Docking

The study of the antimycobacterial activity of the compounds was carried out based on the previously described procedure [29]. For details, see Supporting Information.

## 4. Conclusions

In summary, we have suggested an original procedure for the synthesis of novel 4-alkyl-4*H*-thieno[2′,3′:4,5]pyrrolo[2,3-*b*]quinoxaline derivatives, which are similar to antivirals and antibacterials of the indolo[2,3-*b*]quinoxaline family, through intramolecular oxidative cyclodehydrogenation of the corresponding *N*-alkyl-2-(quinoxaline-2-yl)thiophen-3-amines. The antimycobacterial activity of these derivatives has been estimated for the first time. Molecular docking data demonstrate that 4-alkyl-4*H*-thieno[2′,3′:4,5]pyrrolo[2,3-*b*]quinoxalines appear to be inhibitors of adenosine kinase (*Rv2202c*). The authors believe that the obtained results can be useful for the development of novel antibacterials against various mycobacterial strains.

## Figures and Tables

**Table 1:** Optimization of conditions for the synthesis of *N*^1^,*N*^1^-dimethyl-*N*^2^-(2-(quinoxalin-2-yl)thiophen-3-yl)ethane-1,2-diamine (**2a**).

№	Pd Catalyst (0.1 Equiv.)	Ligand (0.2 Equiv.)	Base (2.5 Equiv.)	Solvent	Reaction Time, Temperature	Reaction Mixtures ^a^ GC–MS (%)
1	Pd(OAc)_2_	Xantphos	*t*-BuONa	toluene	15 min, 150 °C	**2a**—91 **1a**—0 **2-ThioQx**—9
2	Pd(OAc)_2_	PCy_3_	*t*-BuONa	toluene	15 min, 150 °C	**2a**—0 **1a**—0 **2-ThioQx**—100
3	Pd(PPh_3_)_4_	-	*t*-BuONa	toluene	15 min, 150 °C	**2a**—0 **1a**—0 **2-ThioQx**—100
4	Pd(OAc)_2_	PPh_3_	*t*-BuONa	toluene	15 min, 150 °C	**2a**—0 **1a**—0 **2-ThioQx**—100
5	Pd(OAc)_2_	DPEPhos	*t*-BuONa	toluene	15 min, 150 °C	**2a**—4 **1a**—90 **2-ThioQx**—2
6	Pd(OAc)	BrettPhos	*t*-BuONa	toluene	15 min, 150 °C	**2a**—2.4 **1a**—94.4 **2-ThioQx**—3.2
7	Pd(OAc)_2_	P(o-Tol)_3_	*t*-BuONa	toluene	15 min, 150 °C	**2a**—2.7 **1a**—86.5 **2-ThioQx**—10.8
8	Pd(OAc)_2_	XPhos	*t*-BuONa	toluene	15 min, 150 °C	**2a**—3.4 **1a**—82.5 **2-ThioQx**—14.1
9	Pd(dba)_2_	-	*t*-BuONa	toluene	15 min, 150 °C	**2a**—7.7 **1a**—72.0 **2-ThioQx**—20.3
10	Pd_2_(dba)_3_	-	*t*-BuONa	toluene	15 min, 150 °C	**2a**—9.8 **1a**—52.3 **2-ThioQx**—37.9
11	Pd(OAc)_2_	*rac*-BINAP	*t*-BuONa	toluene	15 min, 150 °C	**2a**—90.3 **1a**—0 **2-ThioQx**—9.3
12	Pd(OAc)_2_	dppf	*t*-BuONa	toluene	15 min, 150 °C	**2a**—94,4 **1a**—0 **2-ThioQx**—5.6
13	Pd(OAc)_2_	dppf	KF	toluene	15 min, 150 °C	**2a**—0 **1a**—98,3 **2-ThioQx**—1.7
14	Pd(OAc)_2_	dppf	K_2_CO_3_	toluene	15 min, 150 °C	**2a**—0 **1a**—100 **2-ThioQx**—0
15	Pd(OAc)_2_	dppf	DABCO	toluene	15 min, 150 °C	**2a**—0 **1a**—89.3 **2-ThioQx**—10.7
16	Pd(OAc)_2_	dppf	K_3_PO_4_	toluene	15 min, 150 °C	**2a**—8.7 **1a**—91.3 **2-ThioQx**—0
17	Pd(OAc)_2_	dppf	*t*-BuOK	toluene	15 min, 150 °C	**2a**—40.9 **1a**—0 **2-ThioQx**—59.1
18	Pd(OAc)_2_	dppf	*t*-BuONa	dioxane	15 min, 150 °C	**2a**—80.3 **1a**—0 **2-ThioQx**—19.7
19	Pd(OAc)_2_	dppf	*t*-BuONa	THF	15 min, 150 °C	**2a**—81.7 **1a**—0 **2-ThioQx**—18.3
20	Pd(OAc)_2_	dppf	*t*-BuONa	toluene	15 min, 120 °C	**2a**—94.9 **1a**—0 **2-ThioQx**—5.1
21	Pd(OAc)_2_	dppf	*t*-BuONa	toluene	15 min, 100 °C	**2a**—90.6 **1a**—6.2 **2-ThioQx**—3.2

^a^ The amount of **1a**, **2a**, and **2-ThioQx** in the reaction mixture by GC–MS; **MW**—reactions performed under microwave irradiation; **PCy_3_**—Tricyclohexylphosphine; **Pd(PPh_3_)_4_**—*Tetrakis*(triphenylphosphine)palladium(0); **PPh_3_**—Triphenylphosphine; **DPEPhos**—*Bis*[(2-diphenylphosphino)phenyl]ether; **BrettPhos**—2-(Dicyclohexylphosphino)-3,6-dimethoxy-2′,4′,6′-triisopropyl-1,1′-biphenyl; **P(*o*-tol)_3_**—Tri(*o*-tolyl)phosphine; **XPhos**—2-Dicyclohexylphosphino-2′,4′,6′-triisopropylbiphenyl; **Pd(dba)_2_**—*Bis*(dibenzylideneacetone)palladium(0); **Pd_2_(dba)_3_**—Tris(dibenzylideneacetone)dipalladium(0); ***rac*-BINAP**—(±)-2,2′-*Bis*(diphenylphosphino)-1,1′-binaphthalene; **Xantphos**—4,5-*Bis*(diphenylphosphino)-9,9-dimethylxanthene; **dppf**—1,1′-Ferrocenediyl-*bis*(diphenylphosphine).

**Table 2:** In vitro antimycobacterial activity and cytotoxicity against Vero cells—data for indolo[2,3-*b*]pyrazine derivatives (**7**–**11**).

Entry	Compound	Antimycobacterial Activity Against *Mycobacterium tuberculosis* H_37_Rv (MIC) ^a^		IC_50_ (μg/mL)
		μg/mL	μM	
1	**7a**	25	84.35	n.d
2	**8a**	>25	>80.54	n.d.
3	**9a**	25	93.51	n.d.
4	**10a**	>25	>88.85	n.d.
5	**11a**	>25	73.87	n.d.
6	**7b**	12.5	38.53	11.8 ± 1.4
7	**8b**	25	73.86	n.d
8	**9b**	>25	>84.63	n.d.
9	**10b**	>25	80.79	n.d.
10	**11b**	>25	68.22	n.d.
13	**INH**	0.06	4.38	n.d.

^a^ These results were obtained in three independent experiments. The MIC values were the same in all three experiments, so the standard deviation is not given (equal to 0). n.d.—not determined; INH—Isoniazid.

**Table 3:** Targets of *Mycobacterium tuberculosis* H_37_Rv selected for reverse docking.

Entry	PDB ID	Protein (Gene)
1	1z6k	Citrate lyase (*citE*)
2	1kpg, 1kph	Cyclopropane synthase (*cmaA1*)
3	1kpi	Cyclopropane synthase (*cmaA2*)
4	6ddp, 6nnh, 6nni	Dihydrofolate reductase (*dfrA*)
5	1zlj	DosR regulator protein (*DosR*)
6	2qo1	*β*-Ketoacyl-ACP synthase (*fabH*)
7	5hm3	Acyl-AMP ligase (*fadD32*)
8	5zue	Filamentation temperature-sensitive protein (*ftsZ*)
9	5ecv, 5h8u, 5t8g	Malate synthase (*glcB*)
10	4tvm	Citrate synthase (*gltA2*)
11	5dql	Isocitrate lyase (*icl1*)
12	6ee1	Isocitrate lyase (*icl2*)
13	2isy	Iron-dependent regulator (*IdeR*)
14	4ohu, 4tzk, 5g0t	Enoyl-ACP reductase (*inhA*)
15	5ld8, 6p9l, 6p9m	*β*-Ketoacyl-ACP synthase (*kasA*)
16	2gp6	*β*-Ketoacyl-ACP synthase (*kasB*)
17	2a8x, 4m52	Dihydrolipoyl dehydrogenase (*lpdC*)
18	1uzn	*β*-Ketoacyl-ACP reductase (*mabA*)
19	4qij	1,4-Dihydroxy-2-naphthoate-coenzyme A synthase (*menB*)
20	6o0j	2-Succinyl-5-enolpyruvyl-6-hydroxy-3-cyclohexene-1-carboxylate synthase (*menD*)
21	1tpy	Cyclopropane synthase (*mmaA2*)
22	3ha5	S-Adenosylmethionine-dependent methyltransferase (*mmaA4*)
23	4ewl	*N*-Acetyl-1-D-myo-inosityl-2-deoxy-α-D-glucopyranoside deacetylase (*mshB*)
24	1ozp, 1p0h	Mycothiol synthase (*mshD*)
25	1mop, 1n2h, 4fzj	Pantothenate synthetase (*panC*)
26	6p02, 6oyy, 6oz8	Aspartate decarboxylase (*panD*)
27	1q9j	Phthiocerol dimycocerosyl transferase (*papA5*)
28	1l1e	Cyclopropane synthase (*pcaA*)
29	4n9w, 4nc9	Phosphatidyl mannosyltransferase (*pimA*)
30	6c4q	Polyketide synthase (*pks13*)
31	5xnx	RelA protein (*relA*)
32	6c67	Adenosine kinase (*Rv2202c*)
33	2byo	Lipoprotein LppX (*Rv2945c*)
34	5uhb	Transcription initiation complex (*Rv3457c*)
35	4unr	Thymidylate kinase (*tmk*)

**Table 4:** Docking scores and binding energies of compounds **7**–**11** relative to the cavity of *Rv2202c*.

Ligand	Scores	ΔG_bind_, kJ/mol
**7a**	33.44	−34.49
**8a**	33.53	−34.81
**9a**	34.14	−34.01
**10a**	34.62	−34.90
**11a**	33.48	−37.41
**7b**	34.09	−36.81
**8b**	34.53	−37.53
**9b**	29.5	−36.01
**10b**	32,23	−36.71
**1** **1b**	30.14	−30.47
**Cognate ligand (*6c67*)**	21.46	−26.76

## Data Availability

The original research data is available upon request.

## Supplementary Materials

The following supporting information can be downloaded at: https://www.mdpi.com/article/10.3390/ijms26010369/s1.
