# Supplementary material for: 4-Alkyl-4H-thieno[2′,3′:4,5]pyrrolo[2,3-b]quinoxaline Derivatives as New Heterocyclic Analogues of Indolo[2,3-b]quinoxalines: Synthesis and Antitubercular Activity
Source: Int J Mol Sci. 2025 Jan 3;26(1):369. doi: 10.3390/ijms26010369 (PMC11720412; doi:10.3390/ijms26010369)
Supplement: Supplementary file 1 [file ijms-26-00369-s001.zip › ijms-3390955-supplementary.pdf]

Supporting Information

Table of contents

General Information. 2

Antimycobacterial assay. 3

Colorimetric MTT (tetrazolium) assay 3

Experimental for Molecular docking 5

1. Ligands preparation 5

2. Proteins preparation 5

3. Docking procedure 5

^{1}H (500 MHz, $CDCl_3$) and ^{13}C (125 MHz, $CDCl_3$) NMR Spectra of **1b** 6

^{1}H (500 MHz, $CDCl_3$) and ^{13}C (125 MHz, $CDCl_3$) NMR Spectra of **2a**. 7

^{1}H (500 MHz, $CDCl_3$) and ^{13}C (125 MHz, $CDCl_3$) NMR Spectra of **3a**. 8

^{1}H (500 MHz, $CDCl_3$) and ^{13}C (125 MHz, $CDCl_3$) NMR Spectra of **4a**. 9

^{1}H (500 MHz, $CDCl_3$) and ^{13}C (125 MHz, $CDCl_3$) NMR Spectra of **5a**. 10

^{1}H (500 MHz, $CDCl_3$) and ^{13}C (125 MHz, $CDCl_3$) NMR Spectra of **6a**. 11

^{1}H (500 MHz, $CDCl_3$) and ^{13}C (125 MHz, $CDCl_3$) NMR Spectra of **2b** 12

^{1}H (500 MHz, $CDCl_3$) and ^{13}C (125 MHz, $CDCl_3$) NMR Spectra of **3b** 13

^{1}H (500 MHz, $CDCl_3$) and ^{13}C (125 MHz, $CDCl_3$) NMR Spectra of **4b** 14

^{1}H (500 MHz, $CDCl_3$) and ^{13}C (125 MHz, $CDCl_3$) NMR Spectra of **5b** 15

^{1}H (500 MHz, $CDCl_3$) and ^{13}C (125 MHz, $CDCl_3$) NMR Spectra of **6b** 16

^{1}H (500 MHz, $CDCl_3$) and ^{13}C (125 MHz, $CDCl_3$) NMR Spectra of **7a**. 17

^{1}H (500 MHz, $CDCl_3$) and ^{13}C (125 MHz, $CDCl_3$) NMR Spectra of **8a**. 18

^{1}H (500 MHz, $CDCl_3$) and ^{13}C (125 MHz, $CDCl_3$) NMR Spectra of **9a**. 19

^{1}H (500 MHz, $CDCl_3$) and ^{13}C (125 MHz, $CDCl_3$) NMR Spectra of **10a**. 20

^{1}H (500 MHz, $CDCl_3$) and ^{13}C (125 MHz, $CDCl_3$) NMR Spectra of **11a**. 21

^{1}H (500 MHz, $CDCl_3$) and ^{13}C (125 MHz, $CDCl_3$) NMR Spectra of **7b** 22

^{1}H (500 MHz, $CDCl_3$) and ^{13}C (125 MHz, $CDCl_3$) NMR Spectra of **8b** 23

^{1}H (500 MHz, $CDCl_3$) and ^{13}C (125 MHz, $CDCl_3$) NMR Spectra of **9b** 24

^{1}H (500 MHz, $CDCl_3$) and ^{13}C (125 MHz, $CDCl_3$) NMR Spectra of **10b** 25

^{1}H (500 MHz, $CDCl_3$) and ^{13}C (125 MHz, $CDCl_3$) NMR Spectra of **11b** 26

General Information.

All reagents and solvents were obtained from commercial sources and dried by using the standard procedures before use. Solvents for the Buchwald-Hartwig cross-coupling reaction were deoxygenated by bubbling argon for 1h.

The ^{1}H and ^{13}C NMR spectra were recorded on a Bruker AVANCE-500 instrument using Me_4Si as an internal standard. High resolution mass spectrometry was performed using a Bruker maXis Impact HD spectrometer. Melting points were determined on Boetius combined heating stages and were not corrected.

The GC-MS analysis of all samples was carried out using an Agilent GC 7890A MS 5975C Inert XL EI/CI GC-MS spectrometer with a quadrupole mass-spectrometric detector with electron ionization (70 eV) and scan over the total ionic current in the range m/z 20–1000 and a quartz capillary column HP-5MS (30 m × 0.25 mm, film thickness 0.25 mm). Helium served as a carrier gas, the split ratio of the flow was 1 : 50, and the consumption through the column was 1.0 mL min^{-1}; the initial temperature of the column was 40 °C (storage 3 min), programming rate was 10 °C min^{-1} to 290 °C (storage 20 min), the temperature of the evaporator was 250 °C, the temperature of the source was 230 °C, the temperature of the quadrupole was 150 °C, and the temperature of the transition chamber was 280 °C. Solutions of the samples with a concentration of 3-4 mg mL^{-1} were prepared in THF. Samples of 1 mL of the obtained solutions were analyzed.

Column chromatography was carried out using Alfa Aesar silica gel 0.040-0.063 mm (230–400 mesh). The progress of reactions and the purity of compounds were checked by TLC on Sorbfil plates (Russia), in which the spots were visualized with UV light (λ 254 or 365 nm).

Microwave experiments were carried out in a Discover SP unimodal microwave system (CEM, USA) with a working frequency of 2.45 GHz and the power of microwave radiation ranged from 0 to 300 W. The reactions were carried out in a 35 mL reaction tube with the hermetic Teflon cork. The reaction temperature was monitored, using an inserted IR sensor by the external surface of the reaction vessel.

Antimycobacterial assay.

The study of the antimycobacterial activity of the compounds was carried out on the basis of the REMA procedure.[1]

Preparation of *Mycobacterium tuberculosis* (*Mtb*) suspension.

Suspension of *Mtb* with 1.0 McFarland turbidity was prepared (using saline) from a culture of *Mycobacterium tuberculosis* $H_{37}Rv$ in the logarithmic phase of growth on the Löwenstein-Jensen medium. The resulting suspension (50 μL) was transferred into a tube with Middlebrook 7H9 nutrient broth and OADC growth supplement. In the wells of the plates, the resulting suspension (100 μL) was added.

Preparation of dilutions of the test compounds.

Dilutions of the test compounds were prepared using DMSO and sterile distilled water (Isoniazid was dissolved only in water). Weighed portions of testing compounds were dissolved in the calculated volume of DMSO in such a way as to obtain a stock solution with a concentration of 10000 μg /mL. Further, the dilution was carried out using pure DMSO.

Evaluating procedure:

For testing compounds, 97 μL of culture medium and a solution of the test compounds, prepared at concentrations of 1666.7, 833.3, 416.7, 208.3, 104.2, 52.1, 26.0, and 13.0 μg/ml, were added to the wells of a 96-well plate.

Then, *Mtb* suspension (100 μL) was added to the wells of the plates. Thus, the required concentration of test compounds was obtained in the wells of the plate. The DMSO concentration in all wells is 1.5% (vol.). As a positive control, an *Mtb* culture was used without adding compounds and with the addition of DMSO (final concentration 1.5%). Isoniazid was used as a reference drug. The plates were incubated at 37 ° C for 7 days. After the incubation time, 30 μL of Resazurin solution (with the addition of Tween 80) was added to the wells, and the incubation was continued at 37 °C. The result was taken into account after 24, 48, and 72 hours. The minimal inhibitory concentration (MIC) was taken as the minimum concentration of the test compound that prevents the color change of Resazurin.

Colorimetric MTT (tetrazolium) assay

The Vero cell line (ATCC CCL-81) was incubated in a complete DMEM medium with L-glutamine (Biolot, St. Petersburg) containing 10% fetal calf serum (Biolot, St. Petersburg) in a CO2 incubator at 37 °C. Upon reaching a monolayer in the culture flask, the cells were treated with

---

[1](*a*) J. Palomino, A. Martin, M. Camacho, H. Guerra, J. Swings, F. Portaels, Resazurin Microtiter Assay Plate: Simple and Inexpensive Method for Detection of Drug Resistance in *Mycobacterium tuberculosis.* // *Antimicrob. Agents Chemother*. - **2002**. - Vol. 46. - N 8. - P. 2720–2722; (*b*) N. Taneja, J. Tyagi, Resazurin reduction assays for screening of anti-tubercular compounds against dormant and actively growing *Mycobacterium tuberculosis*, *Mycobacterium bovis* BCG and *Mycobacterium smegmatis.* // *J. Antimicrob. Chemother*. - **2007**. - Vol. 60. - P. 288-293.

warm trypsin:versene solution (1:1), the culture was converted from a monolayer to a suspension, 5 mL of a complete nutrient medium was added, the mixture was mixed well and the cells were counted using a Goryaev chamber. Based on the obtained value, a suspension containing 15,000 cells/ml was prepared in a complete nutrient medium, and 200 μL of the resulting suspension was added to the wells of a 96-well plate (3000 cells/well). The plates were incubated for 24 hours in a CO_2 incubator at 37 °C.

A compound **7b** (3 mg) was dissolved in 0.6 ml of DMSO, obtaining a stock solution with a concentration of 5000 μg/mL. Then, dilutions were carried out (using DMSO as a solvent) in such a way as to obtain a series of solutions with the following concentrations of the studied compound: 2500, 625, and 156.3 μg/mL. 3980 μl of a complete nutrient medium and 20 μL of the above-prepared solutions of compound **7b** in DMSO were added to the test tubes. Thus, solutions of the studied compound in a complete nutrient medium were obtained in the following concentrations: 25, 12.5, 3.13, and 0.78 μg/mL. The concentration of DMSO in the obtained solutions was constant (0.5 vol.%).

At the end of the 24-hour incubation, the nutrient medium was removed from the wells of the plates, and 200 μL of the compound **7b** solutions in a complete nutrient medium, prepared as described above, were added to each well. 200 μL of complete nutrient medium was added to the wells of the intact control, and 200 μL of complete nutrient medium with the addition of DMSO (final concentration in the well 0.5% vol.) was added to the wells with DMSO. The plates were incubated for 24 hours in a CO_2 incubator at 37°C.

After 24 hours of incubation, the nutrient medium was removed from the wells of the plates. Then, 200 μL of fresh complete nutrient medium and 50 μL of an aqueous solution of MTT reagent (5 mg/mL) were added to each well. The plates were incubated for 4 hours in a CO_2 incubator at 37 °C. After this incubation period, the nutrient medium was removed from the wells, and 200 μL of DMSO was added. The plate was shaken for ten minutes to dissolve the formazan crystals, and the optical density of the solution in each well was measured at 540 nm using a Multiskan FC plate spectrophotometer (Thermo Scientific).

Statistical analysis of the collected data was conducted using Microsoft Office Excel 16 software. Graphs were created to show the relationship between cell viability and the concentration of the tested compound. For the plate containing compound **7b**, the optical density values from the wells where the cells were incubated in a nutrient medium with a 0.5% DMSO concentration by volume were used as the baseline for 100% viability. The IC_{50} was then calculated as the concentration of the compound that resulted in a 50% reduction in cell viability. All values reported below were obtained from three independent experiments.

Experimental for Molecular docking

1. Ligands preparation

2D Structures of ligands were generated using the ChemDraw panel in ChemBio3D Ultra 14.0. 3D Structures of ligands were generated by ChemBio3D Ultra 14.0. Energy minimization of ligand structures was performed by «MM2 minimize» function implemented in ChemBio3D Ultra 14.0. The SYBYL2 (*.mol2) format of structures were saved and used for docking study.

2. Proteins preparation

The crystal structures of proteins were downloaded from protein data bank (PDB, www.rcsb.org). Proteins preparation for docking was performed using the GOLD Docking Wizard in GOLD suit (2020.0 CSD Release). The proteins preparation included adding hydrogen atoms, deleting of water molecules, deleting ligands (except for cofactors). For proteins available at PDB as several separate structures, two or three different structures were downloaded and superimposed at A-chains in GOLD Docking Wizard to Best-Match Weighting of 10.0 in order to consider protein flexibility.

3. Docking procedure

Ligand-protein docking was performed in GOLD suit (2020.0 CSD Release).

For protein structures having a cognate ligand (substrate or inhibitor), binding site was determined as a space within a radius of 10–20 Å from the cavity of the deleted cognate ligand. For protein structures without a cognate ligand, binding site was predicted by the comparison of docking solutions of all ligands in the hole protein; the place where the ligands were most often located was considered as a binding site, and the final docking procedure for all examined ligands was performed in the space within a radius of 14–17 Å from a selected solvent-accessible atom from the predicted cavity for all ligands.

Ligands were set as flexible with rotatable bonds. Proteins were treated as rigid. For superimposed proteins, ensemble docking was applied to consider possible protein flexibility. ChemScore was used as fitness function. If the top three solutions were within 1 Å, the number of genetic algorithm runs was terminated early. All other parameters and options in GOLD were used as default.

All calculations were carried out in triplicate. Statistical treatment was carried out using GraphPad Prism 8 (8.0.1 release) program. Best docking poses for each ligand were saved as *.pdb files in Hermes. Then protein-ligand interactions were determined by BIOVIA Discovery Studio Visualizer (Discovery Studio 2020 v20.1.0.19295) using default parameters and options.

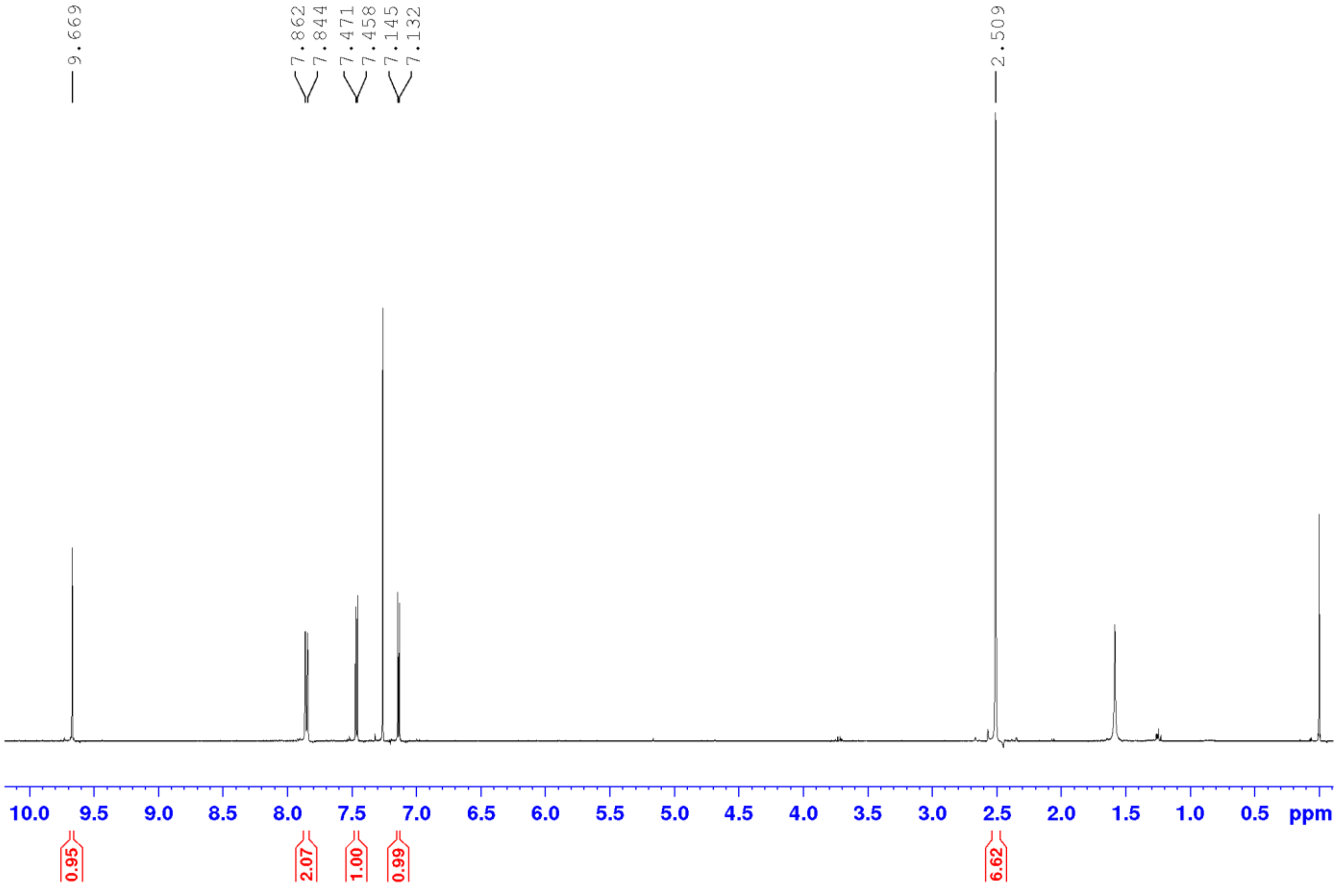

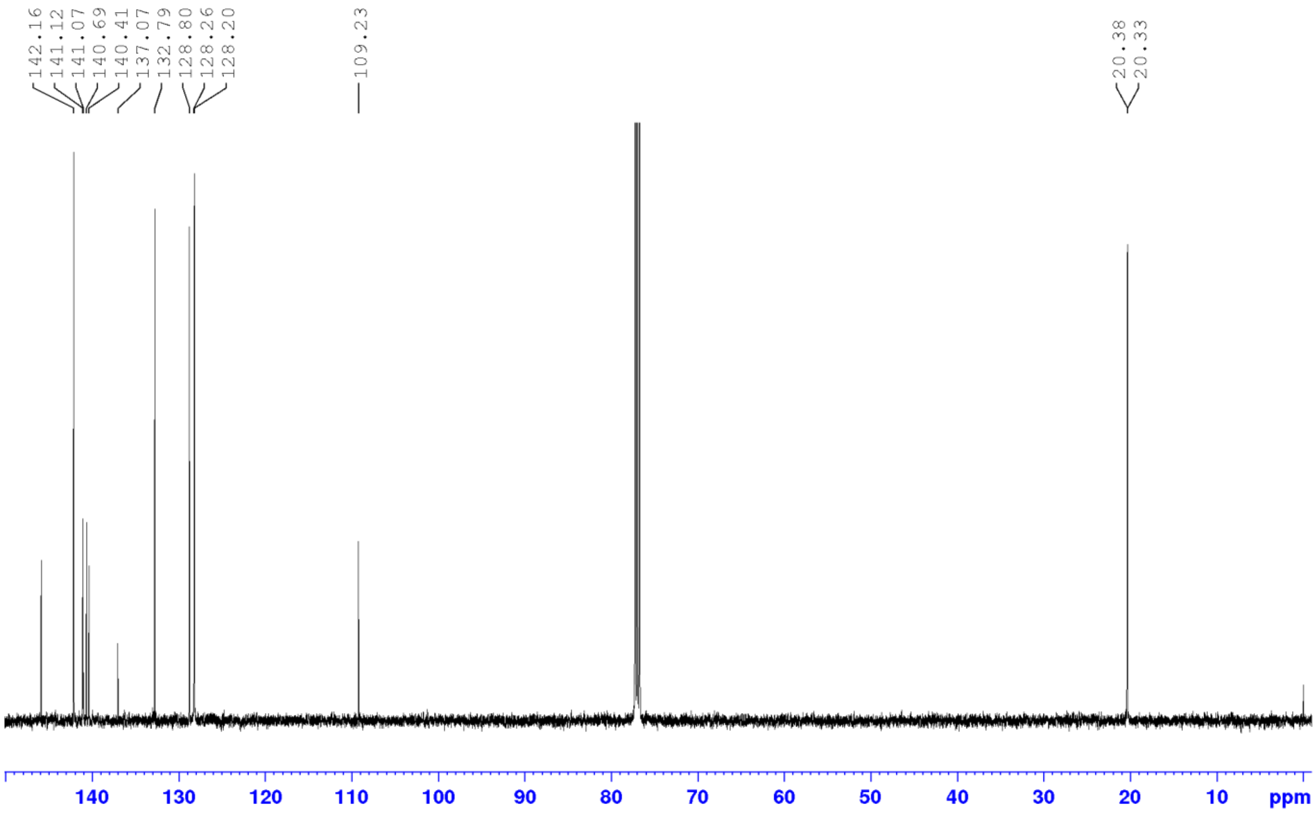

^{1}H (500 MHz, $CDCl_3$) and ^{13}C (125 MHz, $CDCl_3$) NMR Spectra of **1b**.

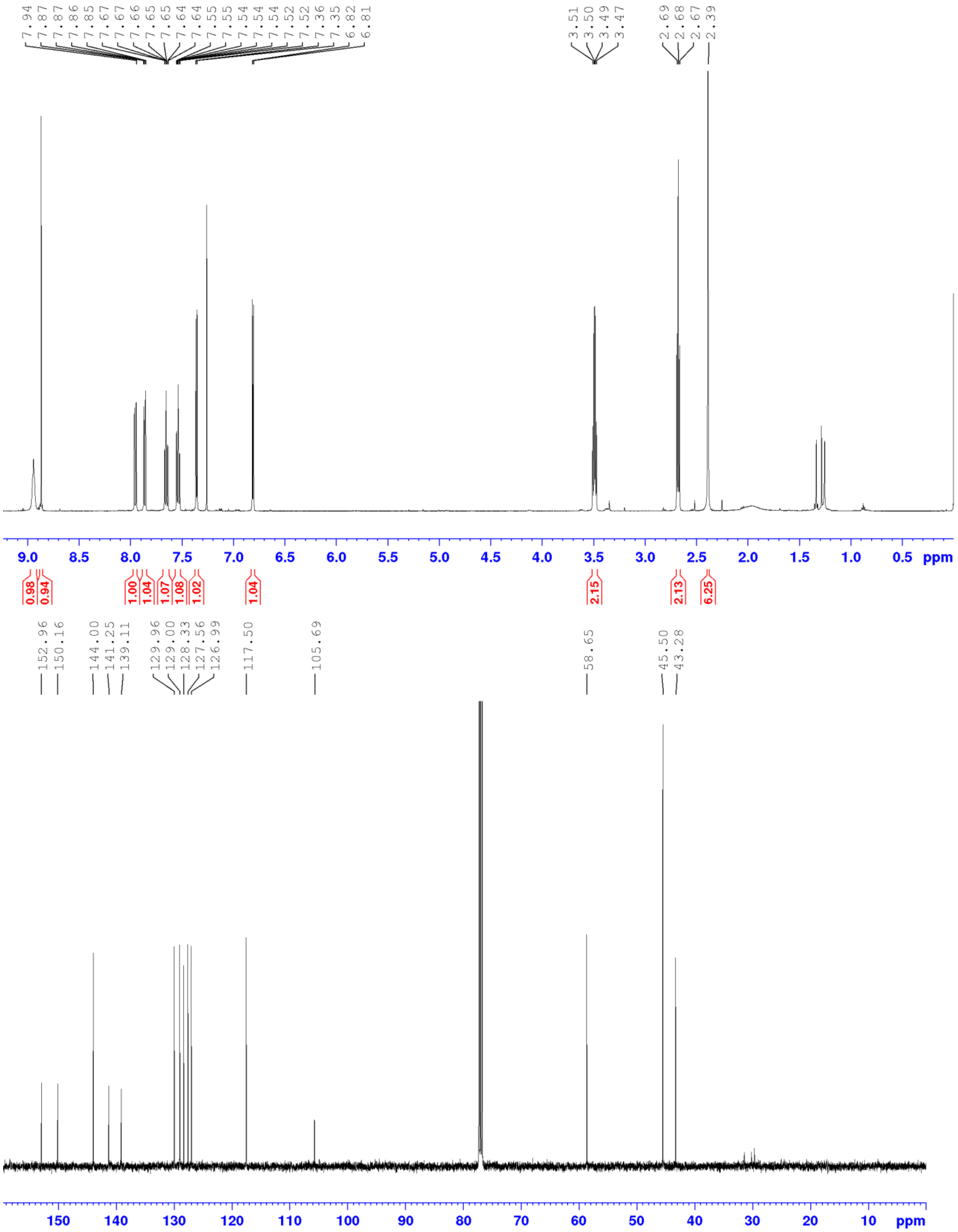

^{1}H (500 MHz, $CDCl_3$) and ^{13}C (125 MHz, $CDCl_3$) NMR Spectra of **2a**.

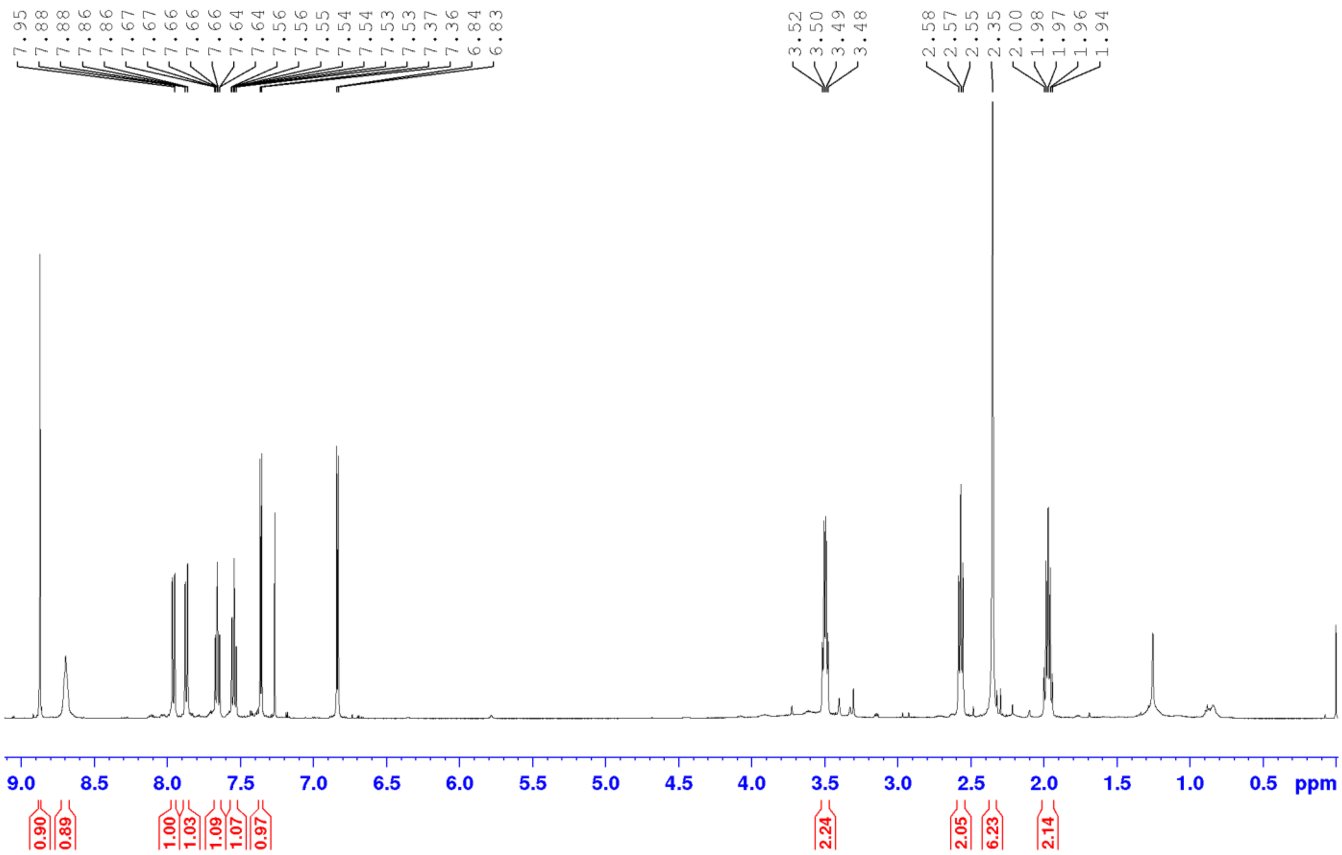

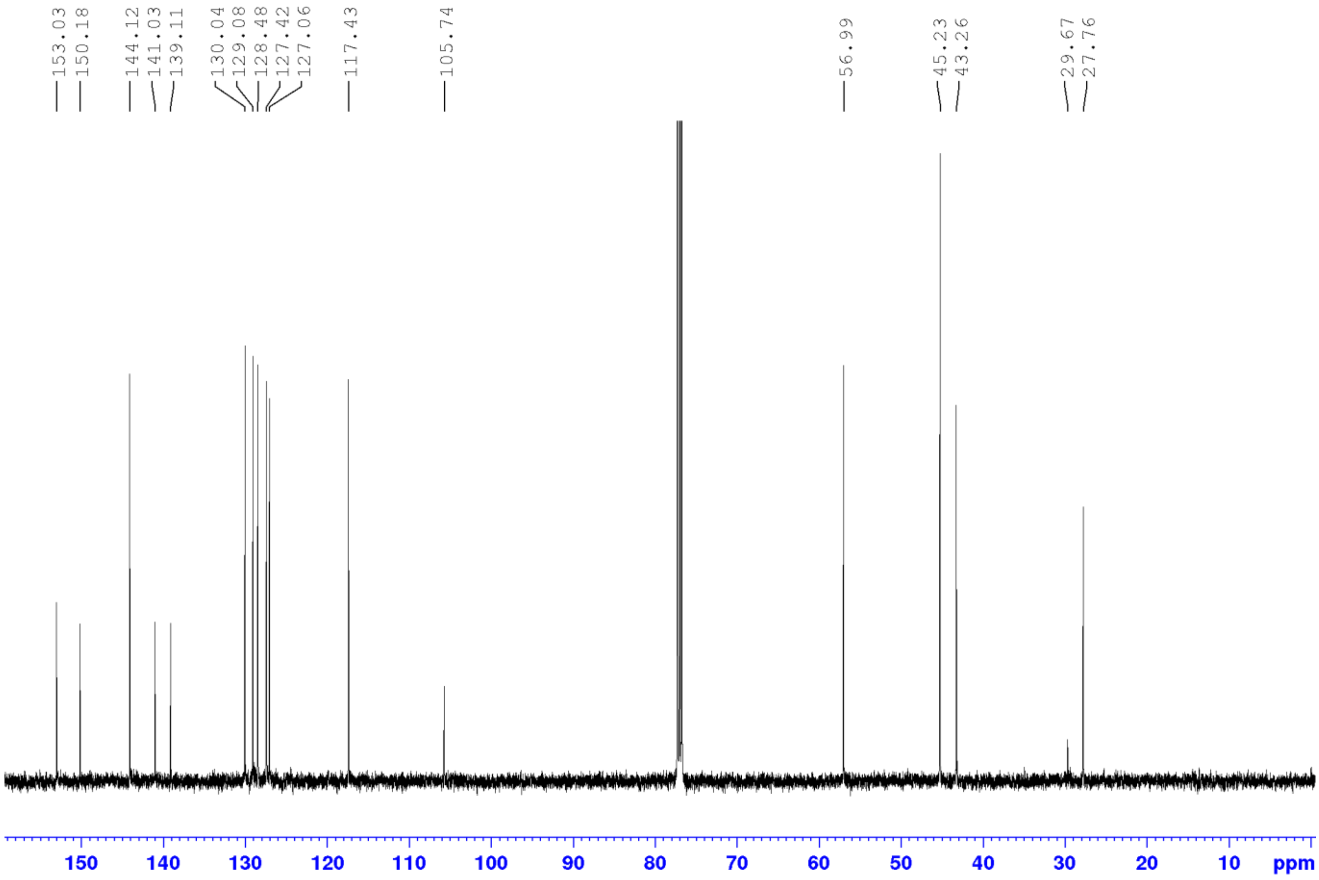

^{1}H (500 MHz, $CDCl_3$) and ^{13}C (125 MHz, $CDCl_3$) NMR Spectra of **3a**.

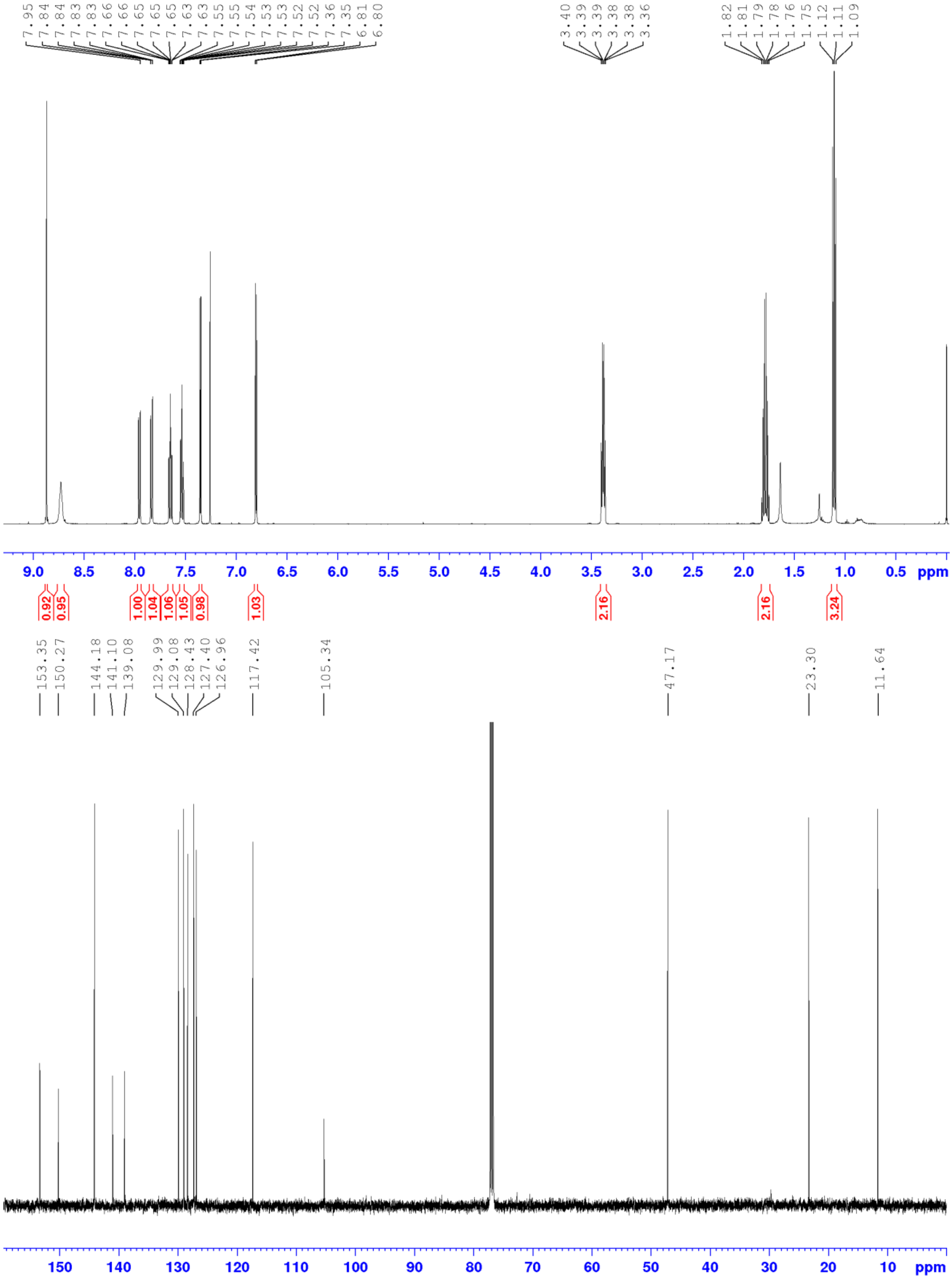

^{1}H (500 MHz, $CDCl_3$) and ^{13}C (125 MHz, $CDCl_3$) NMR Spectra of **4a**.

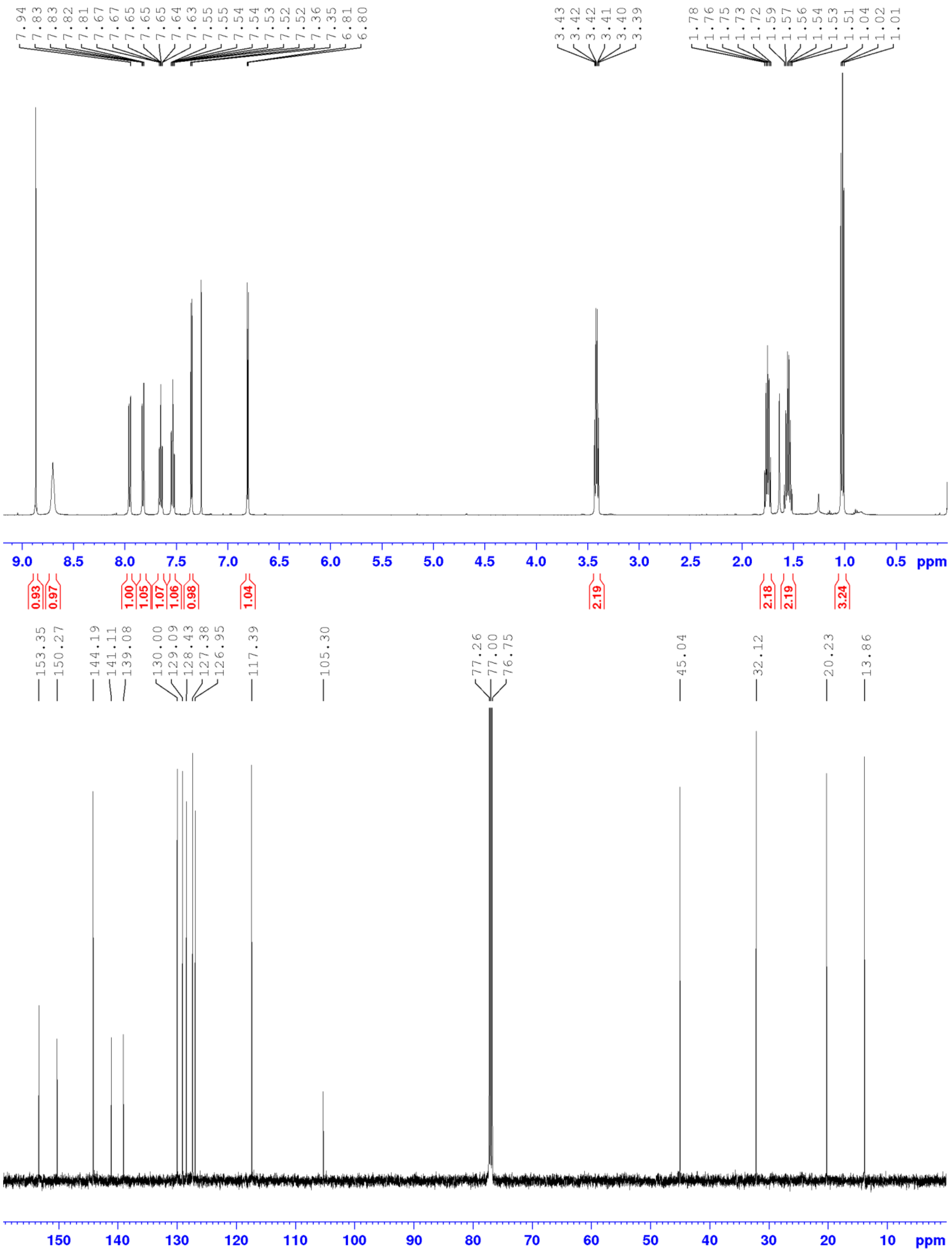

^{1}H (500 MHz, $CDCl_3$) and ^{13}C (125 MHz, $CDCl_3$) NMR Spectra of **5a**.

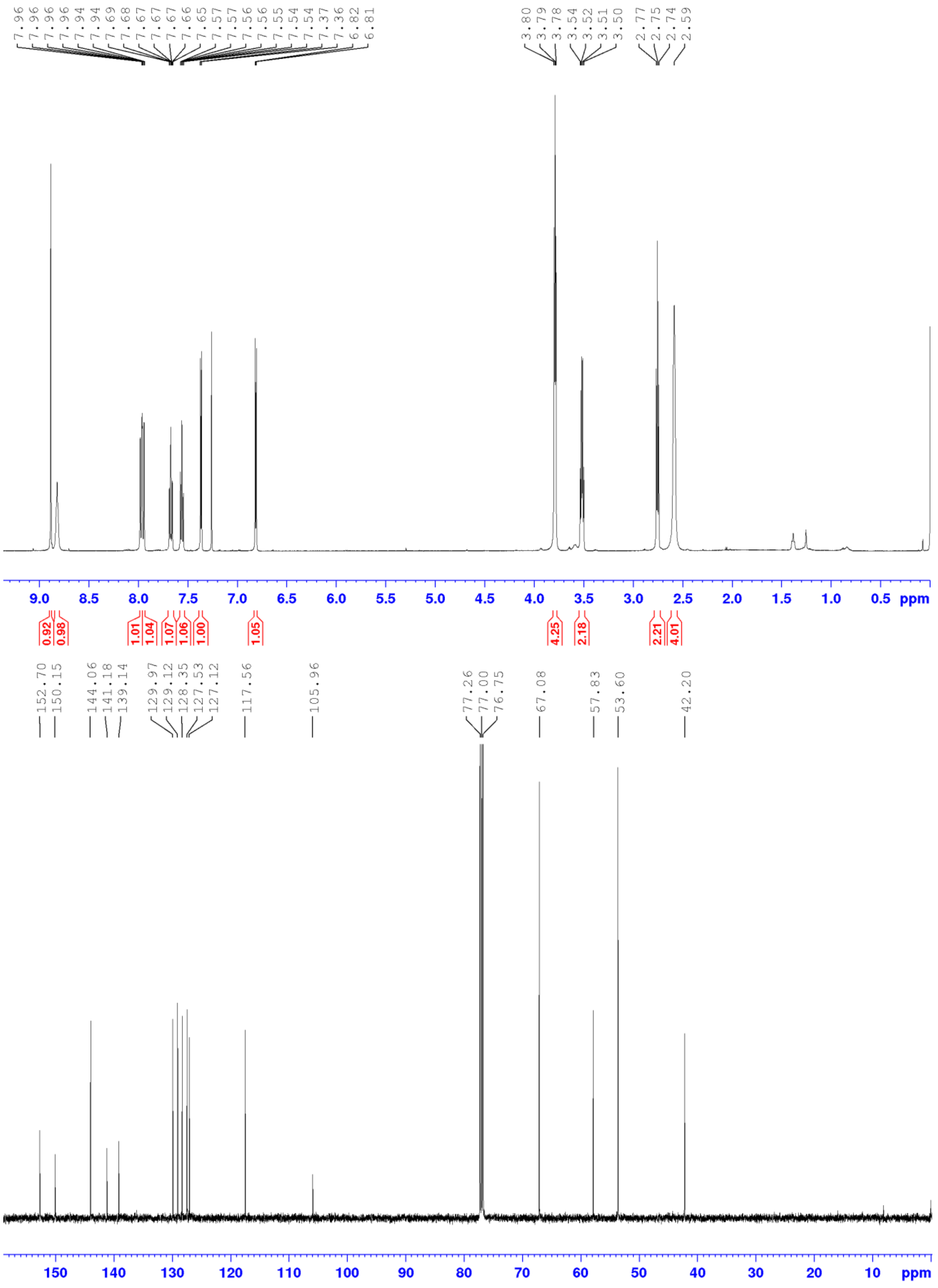

^{1}H (500 MHz, $CDCl_3$) and ^{13}C (125 MHz, $CDCl_3$) NMR Spectra of **6a**.

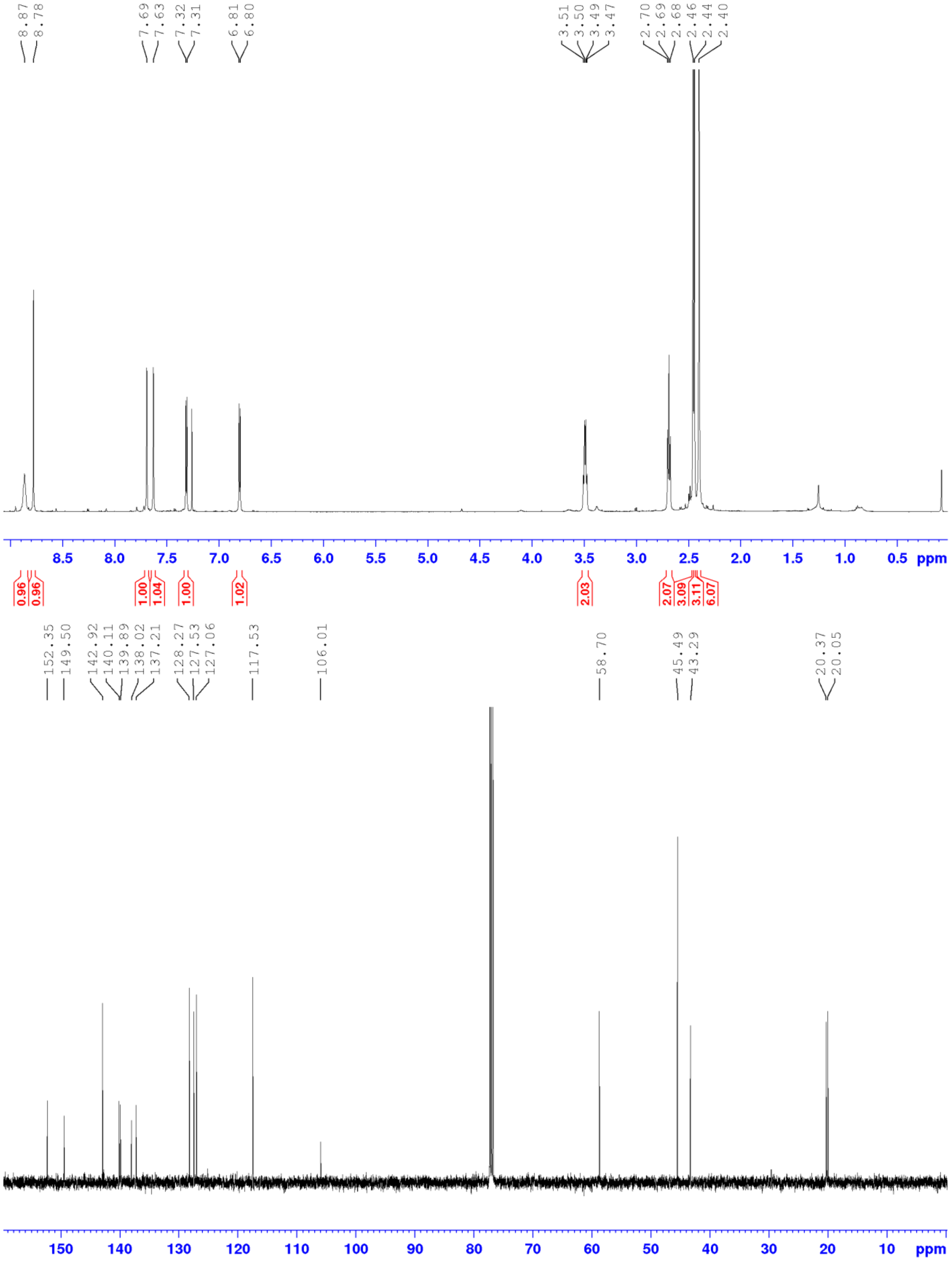

^{1}H (500 MHz, $CDCl_3$) and ^{13}C (125 MHz, $CDCl_3$) NMR Spectra of **2b**.

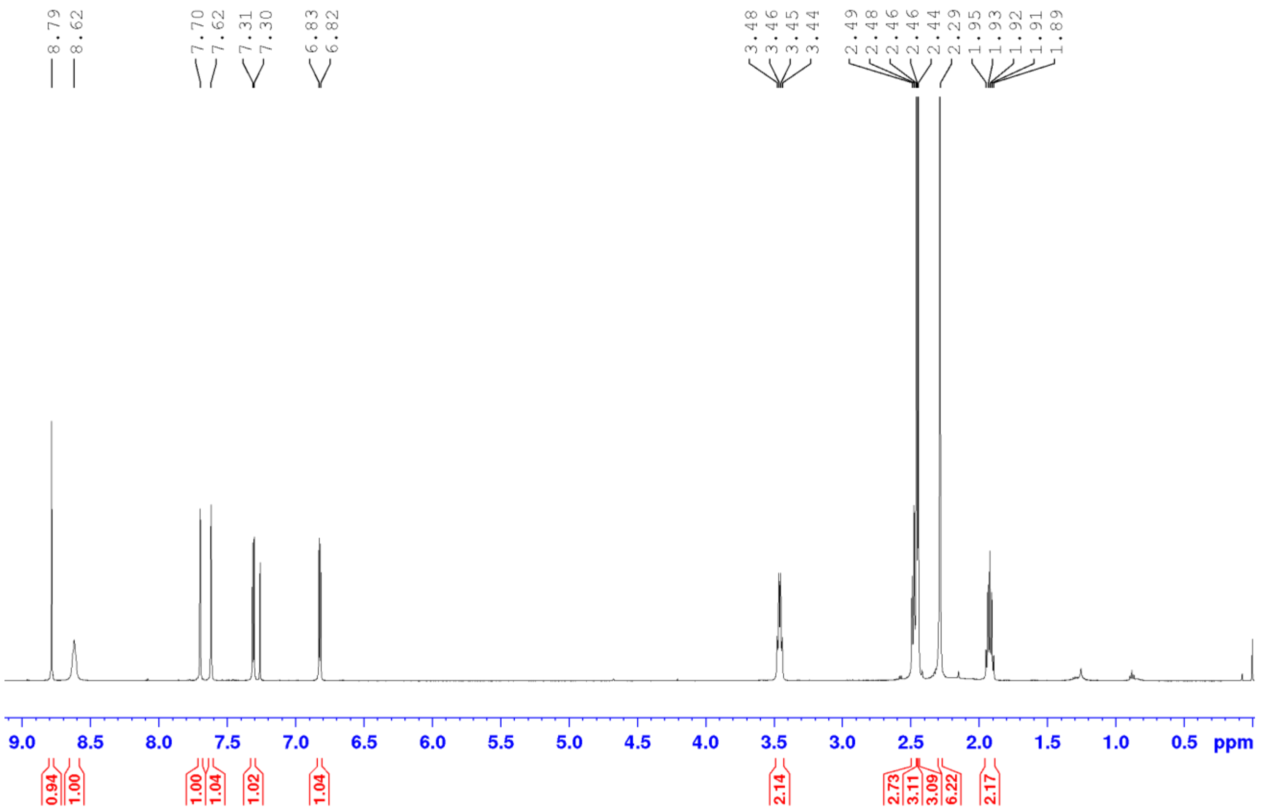

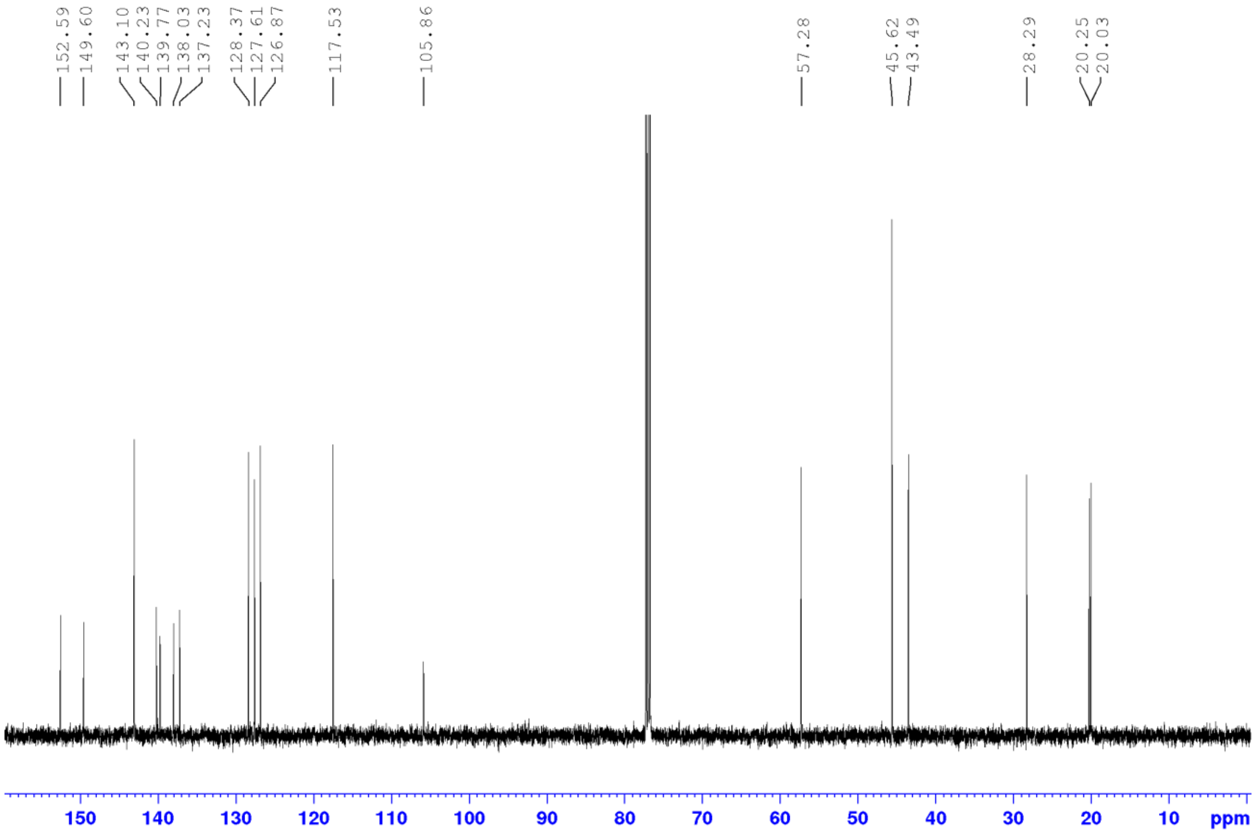

^{1}H (500 MHz, $CDCl_3$) and ^{13}C (125 MHz, $CDCl_3$) NMR Spectra of **3b**.

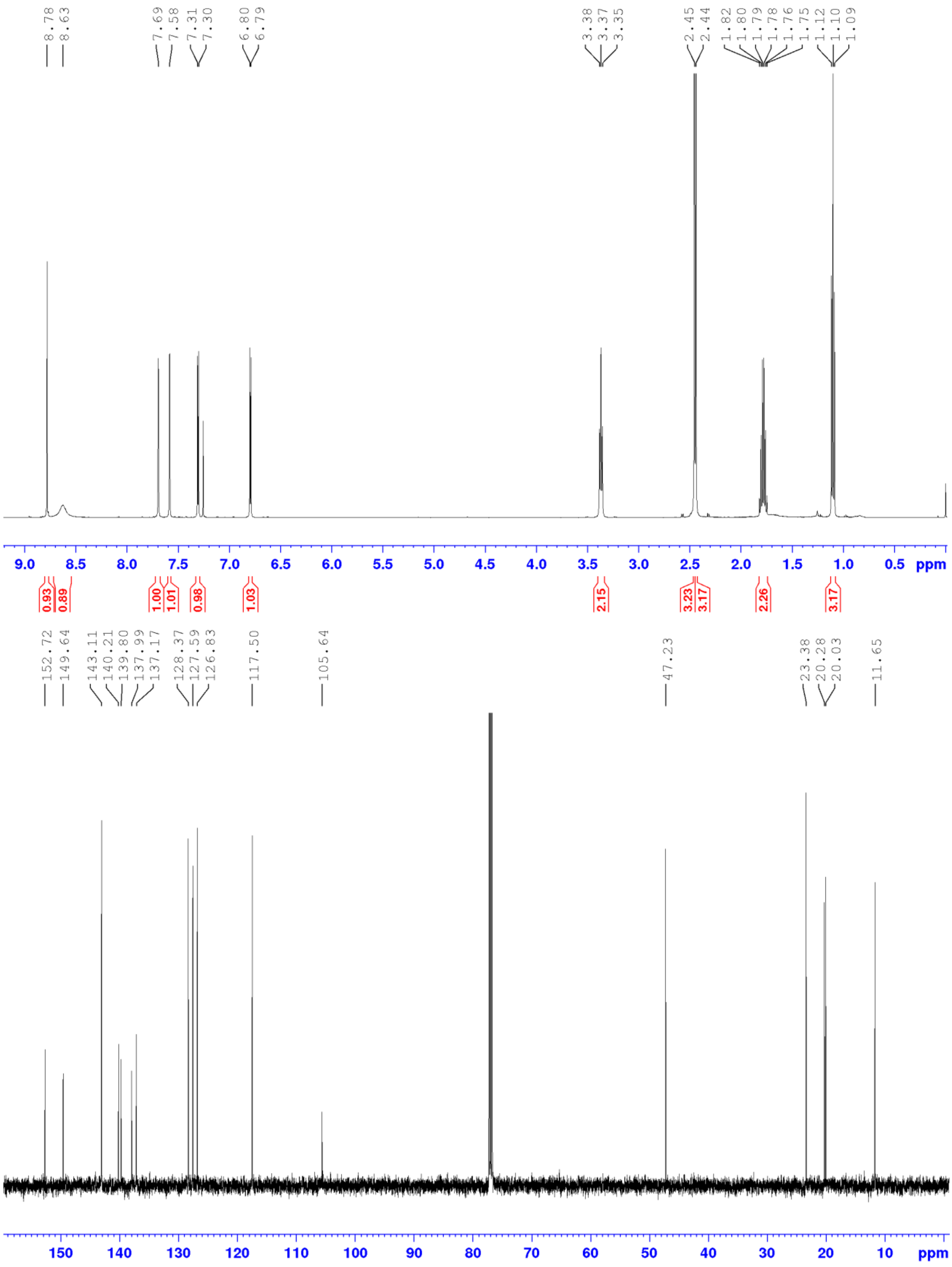

^{1}H (500 MHz, $CDCl_3$) and ^{13}C (125 MHz, $CDCl_3$) NMR Spectra of **4b**.

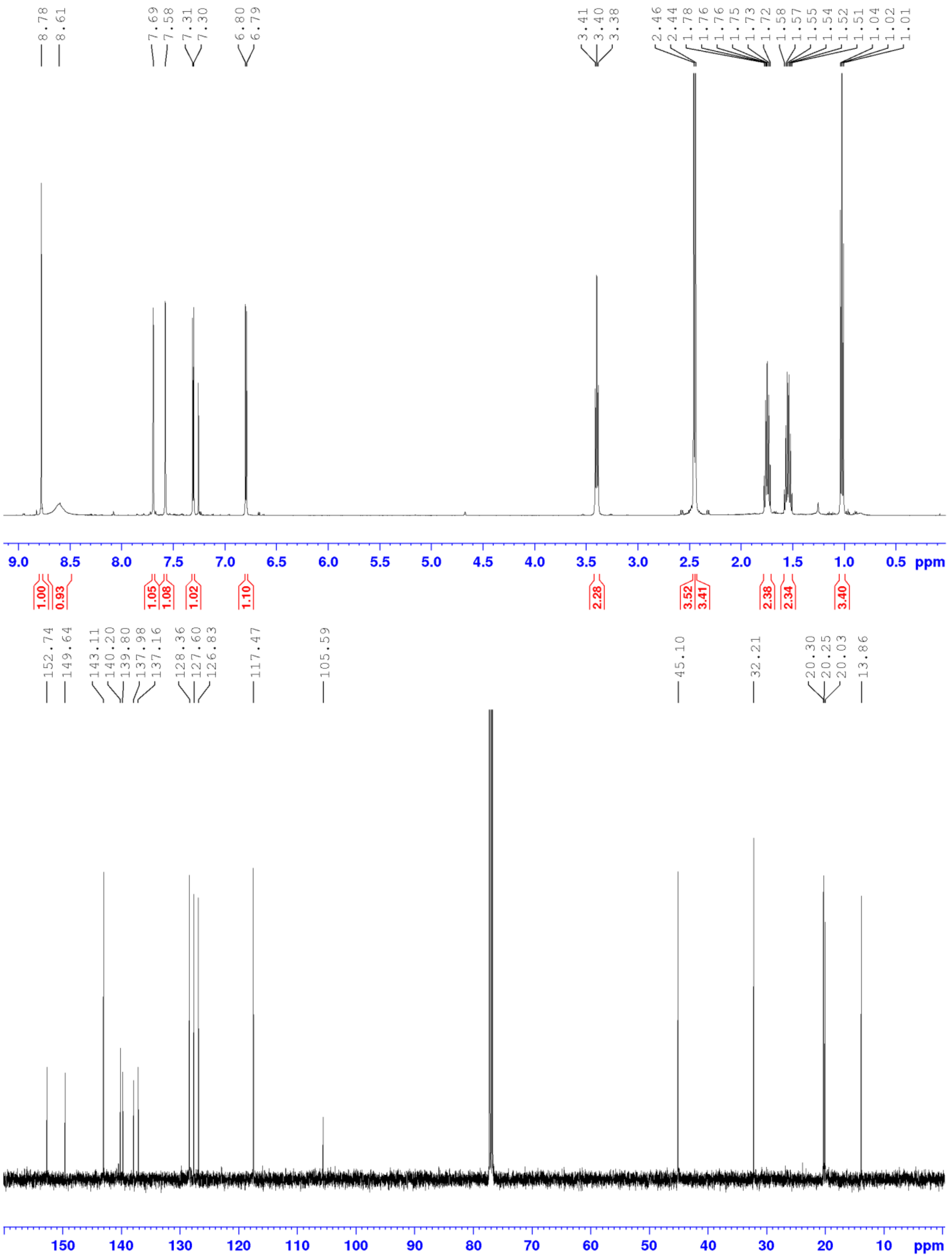

^{1}H (500 MHz, $CDCl_3$) and ^{13}C (125 MHz, $CDCl_3$) NMR Spectra of **5b**.

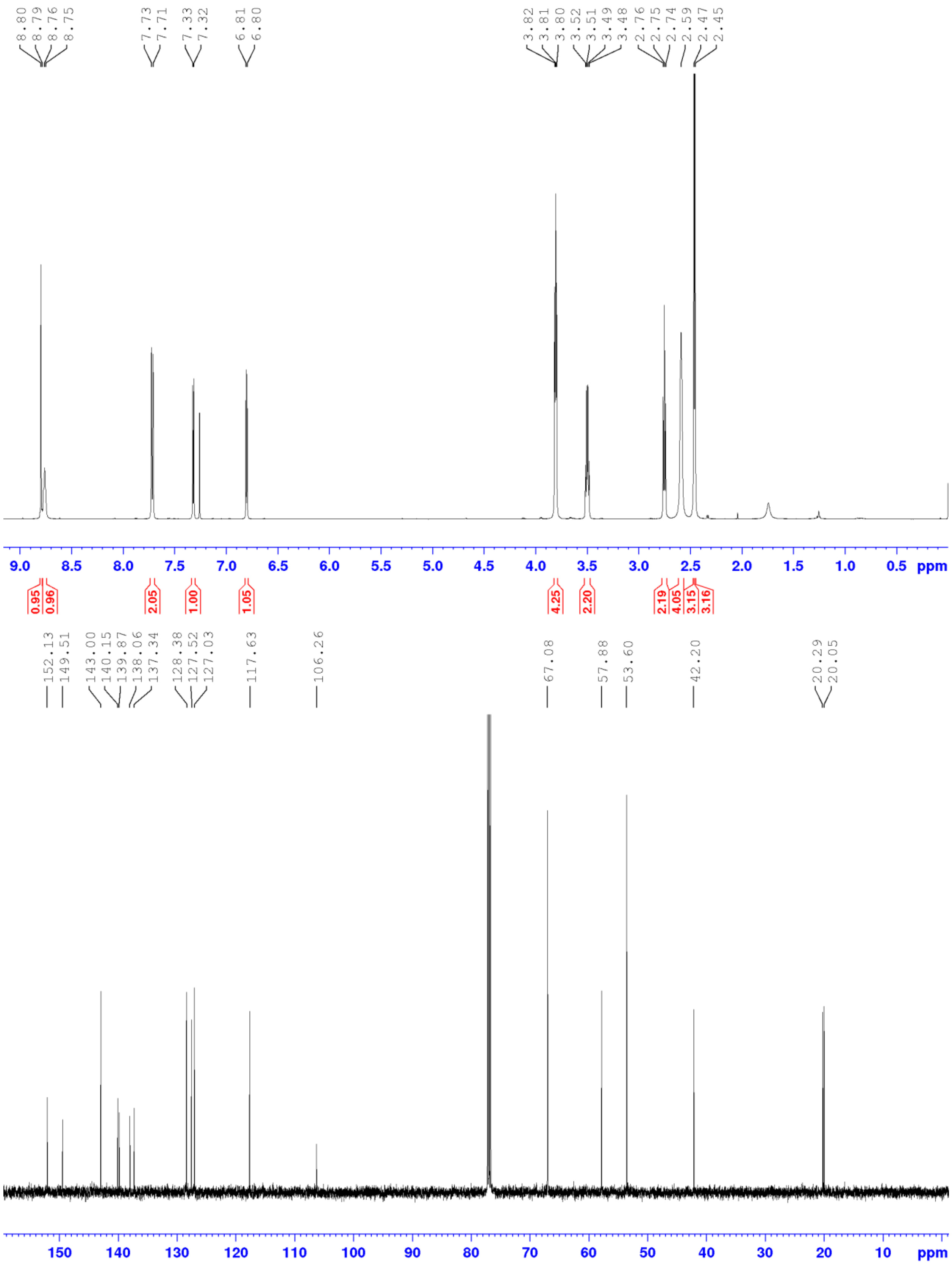

^{1}H (500 MHz, $CDCl_3$) and ^{13}C (125 MHz, $CDCl_3$) NMR Spectra of **6b**.

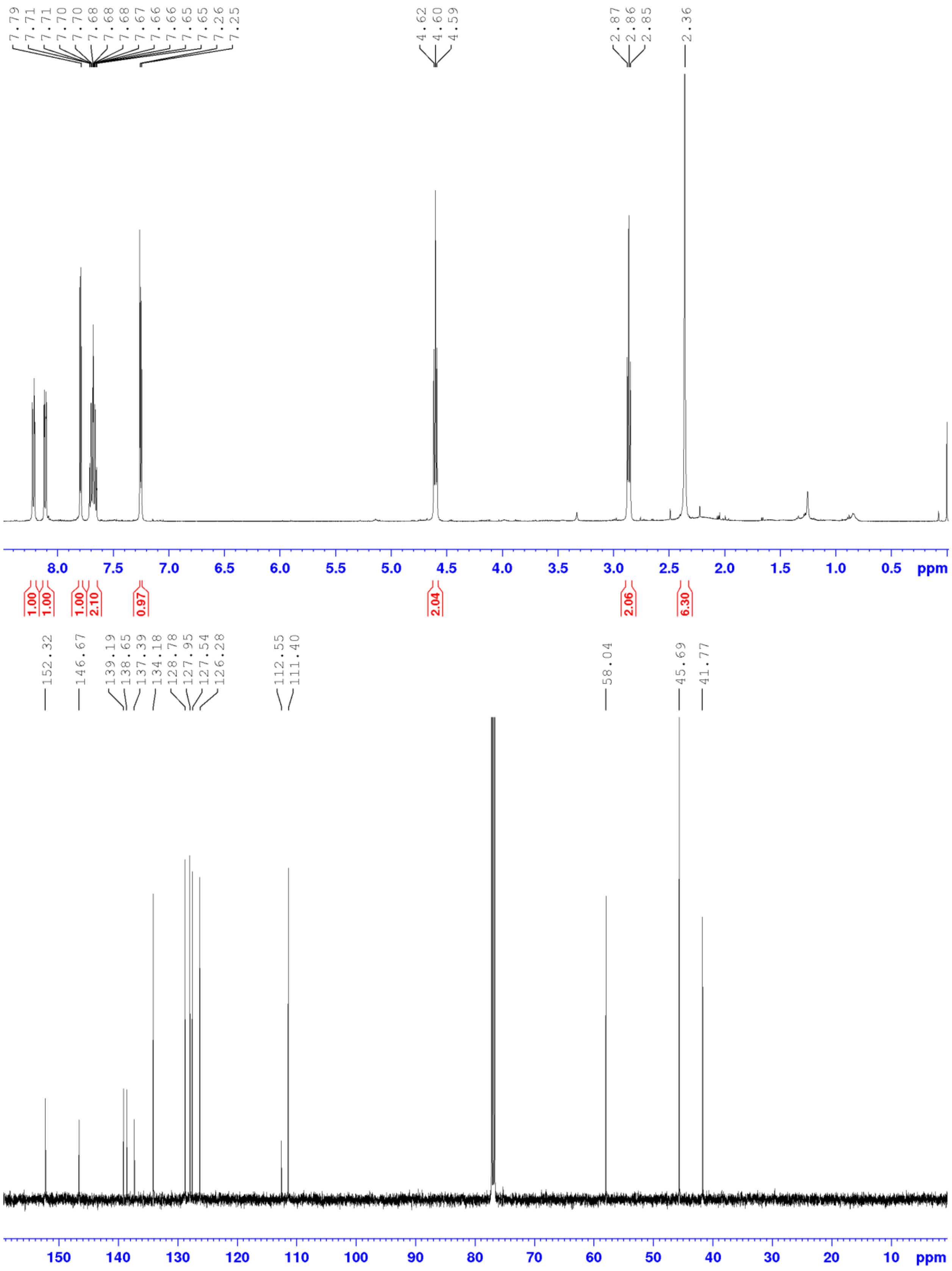

^{1}H (500 MHz, $CDCl_3$) and ^{13}C (125 MHz, $CDCl_3$) NMR Spectra of **7a**.

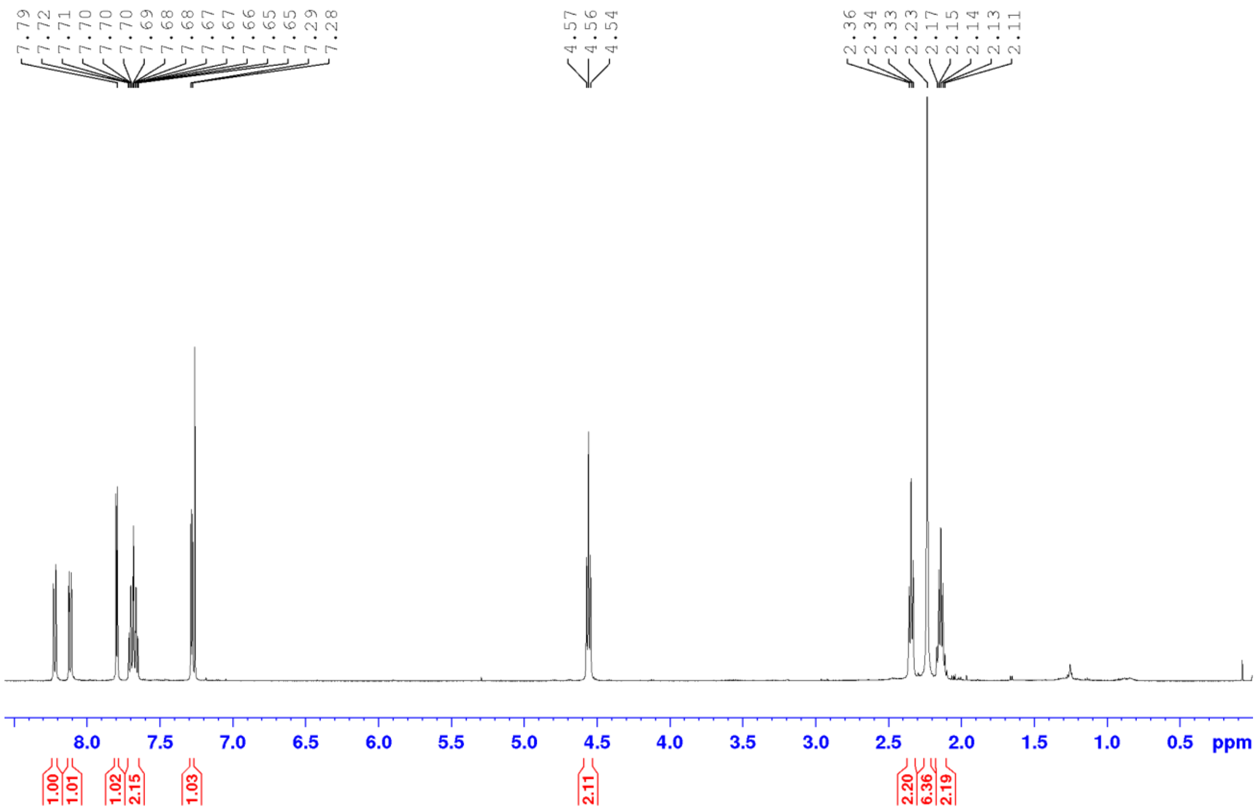

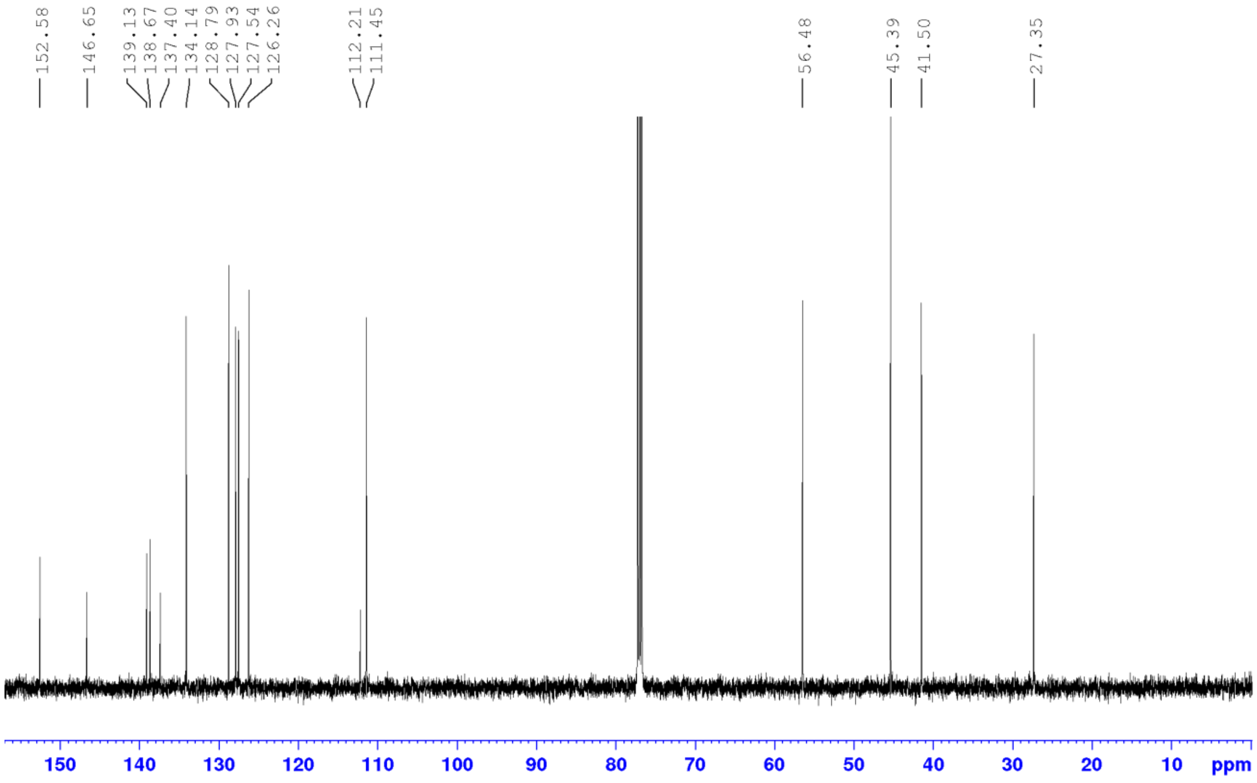

^{1}H (500 MHz, $CDCl_3$) and ^{13}C (125 MHz, $CDCl_3$) NMR Spectra of **8a**.

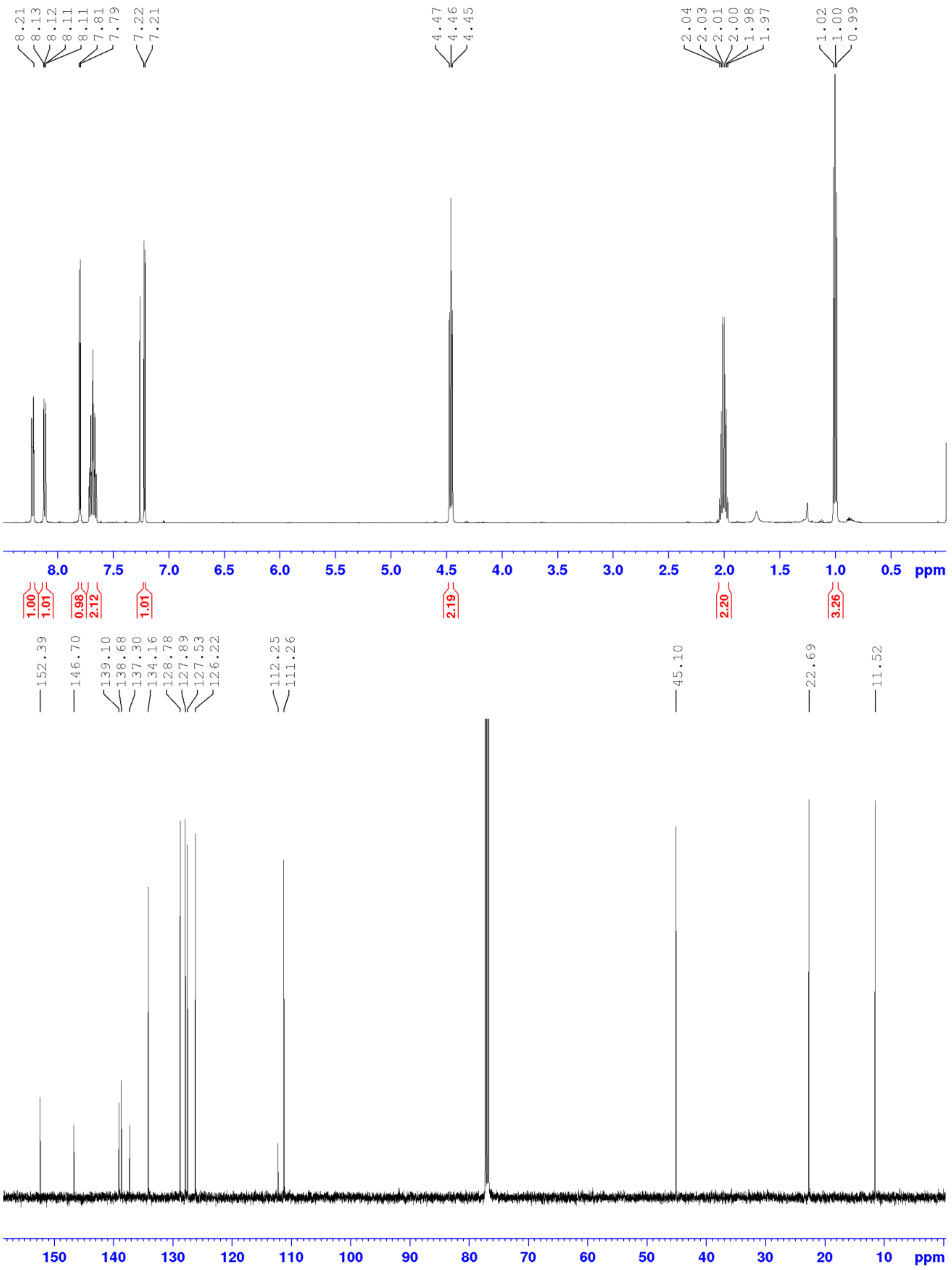

^{1}H (500 MHz, $CDCl_3$) and ^{13}C (125 MHz, $CDCl_3$) NMR Spectra of **9a**.

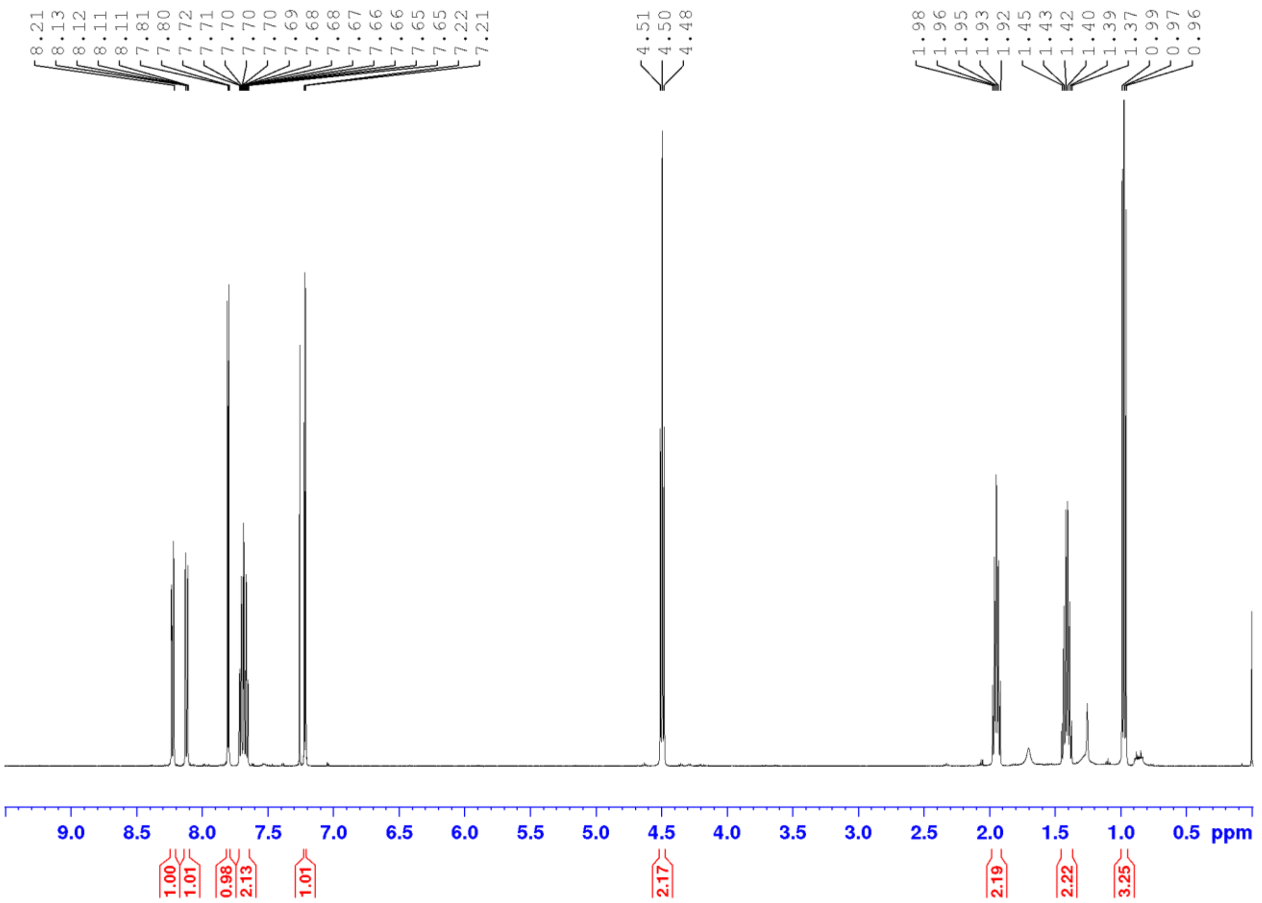

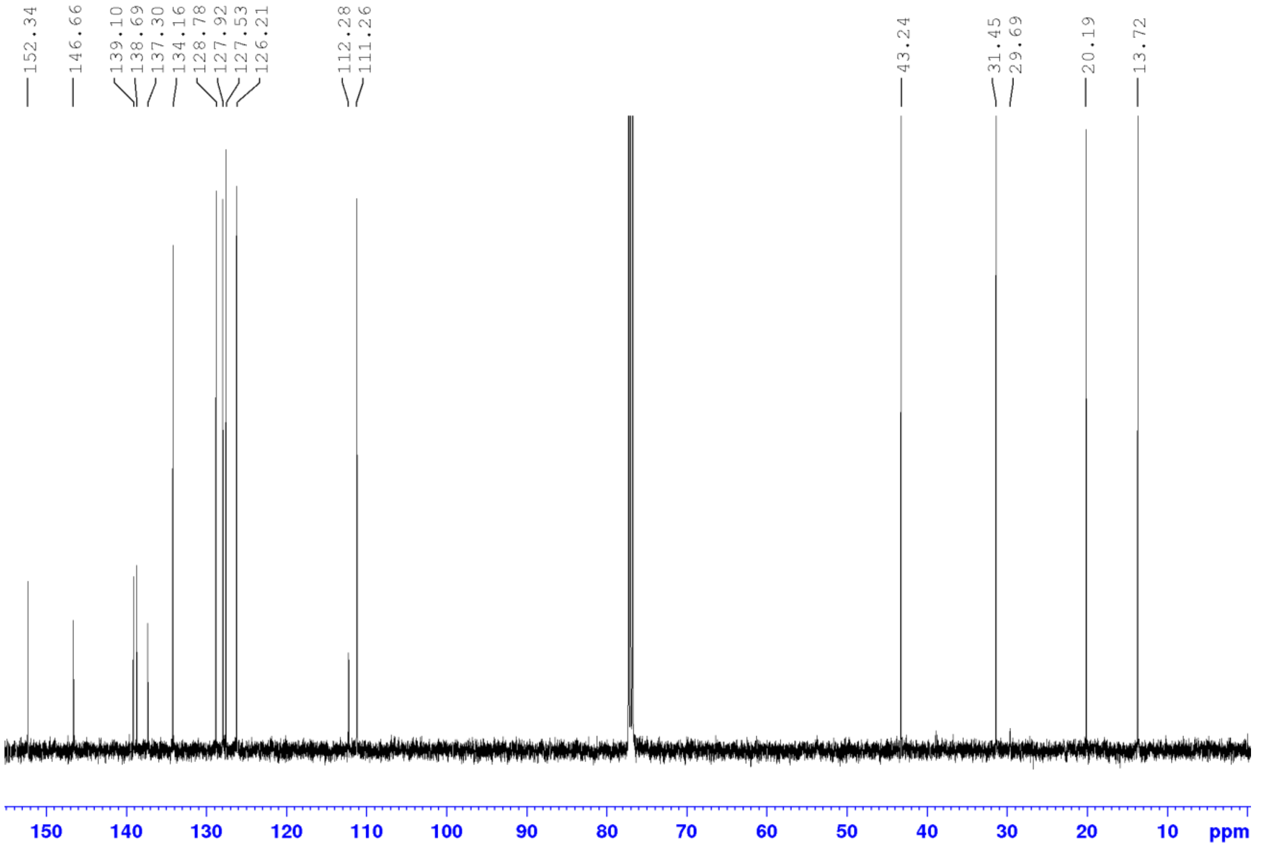

^{1}H (500 MHz, $CDCl_3$) and ^{13}C (125 MHz, $CDCl_3$) NMR Spectra of **10a**.

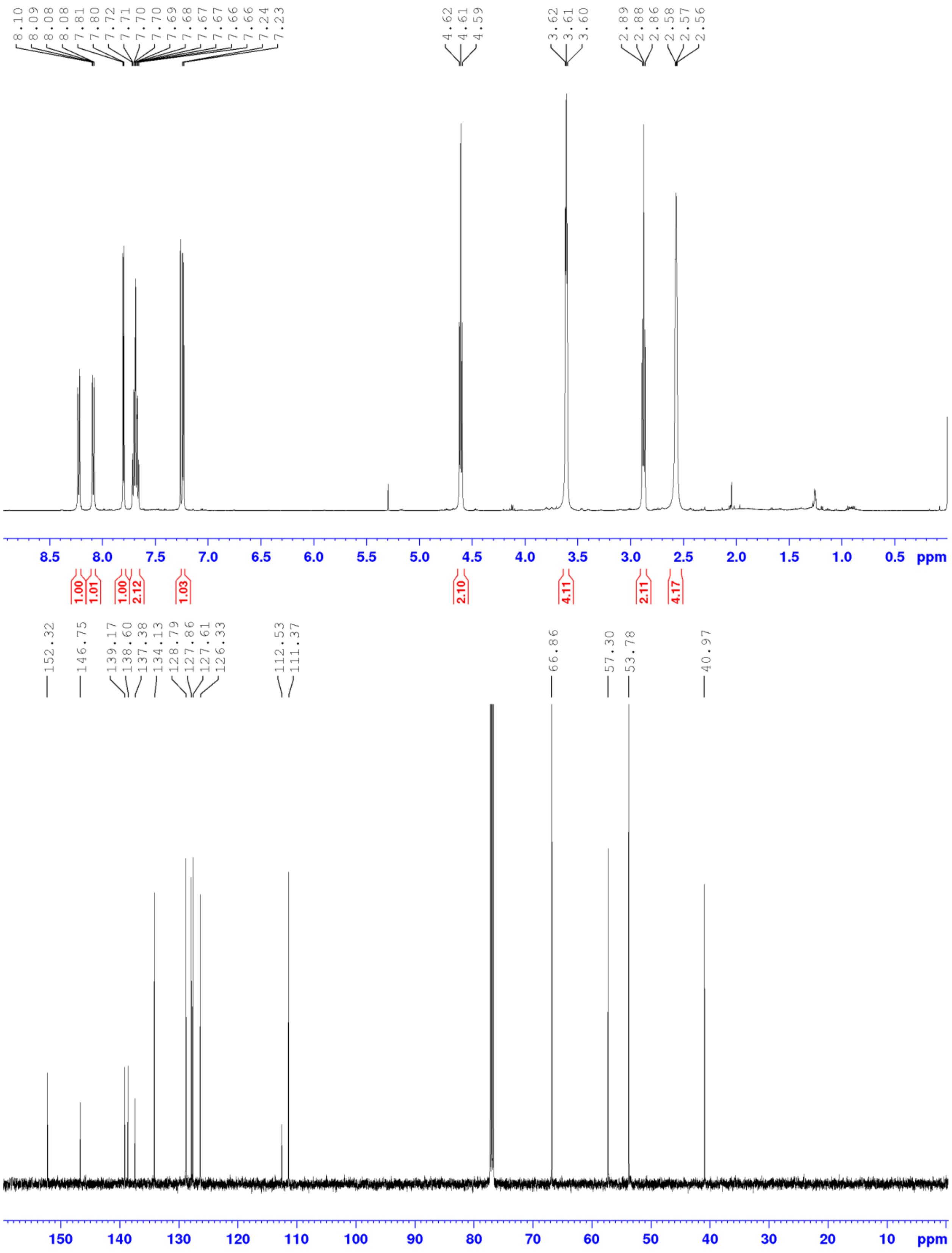

^{1}H (500 MHz, $CDCl_3$) and ^{13}C (125 MHz, $CDCl_3$) NMR Spectra of **11a**.

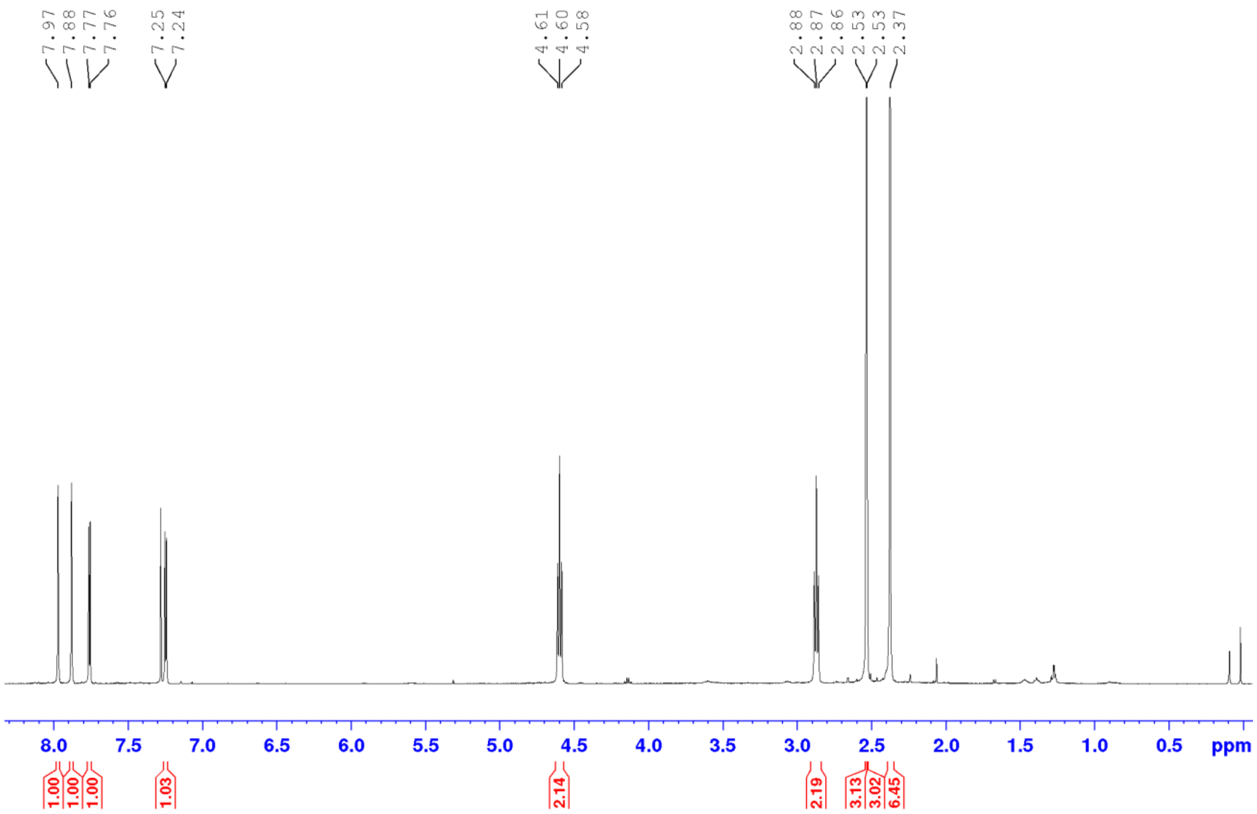

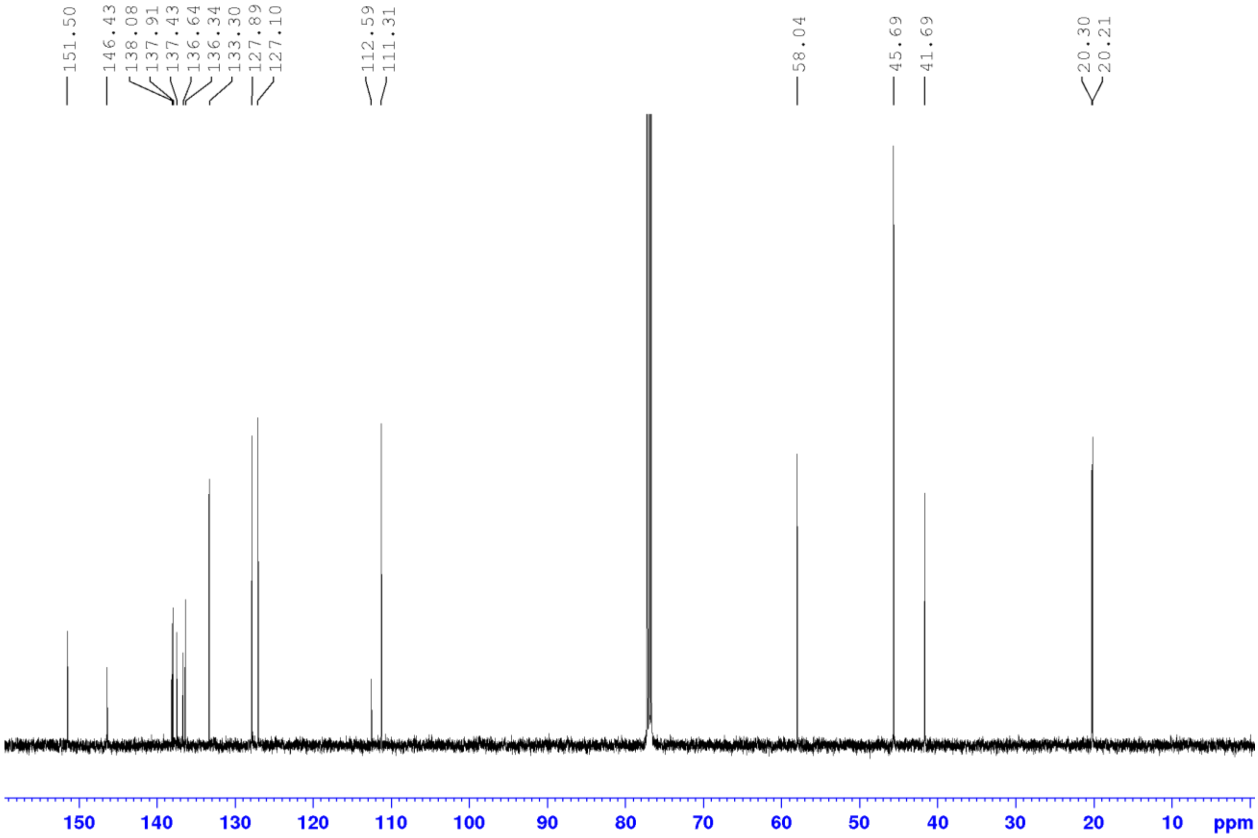

^{1}H (500 MHz, $CDCl_3$) and ^{13}C (125 MHz, $CDCl_3$) NMR Spectra of **7b**.

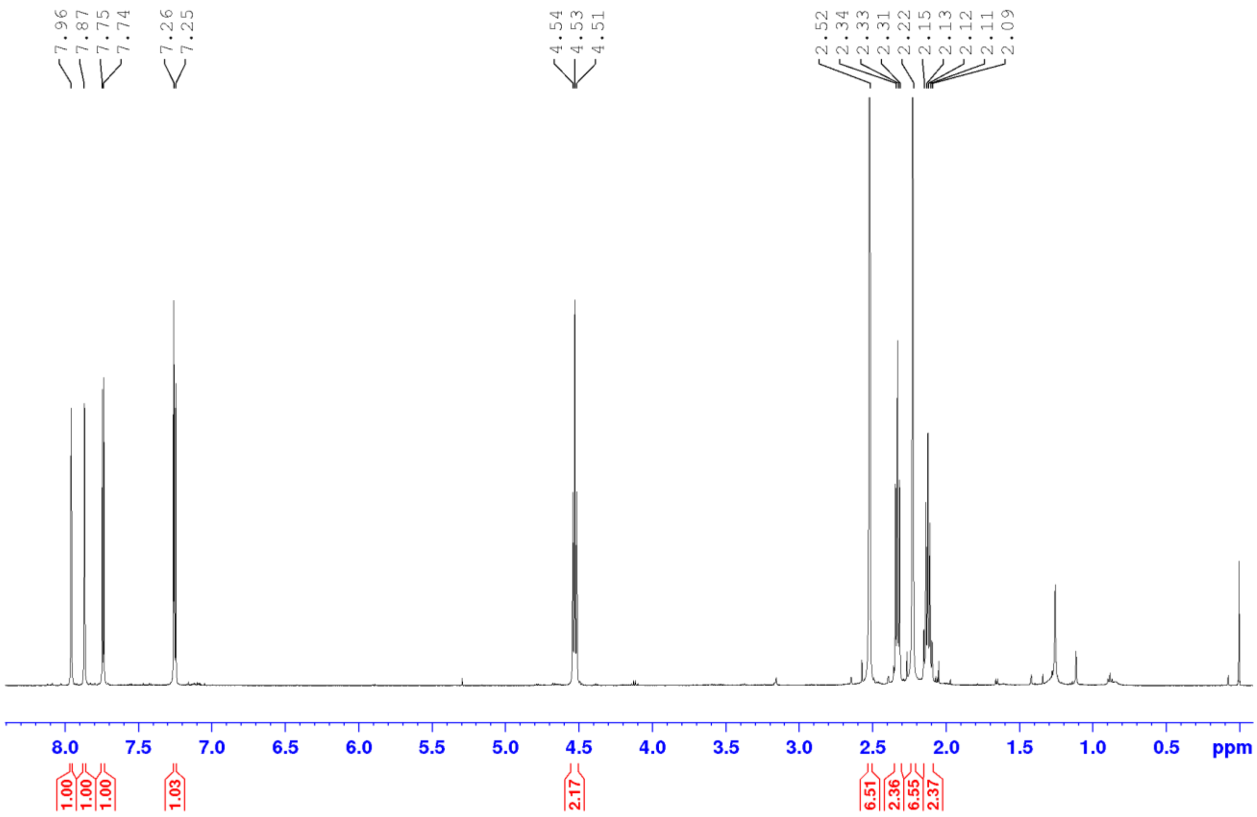

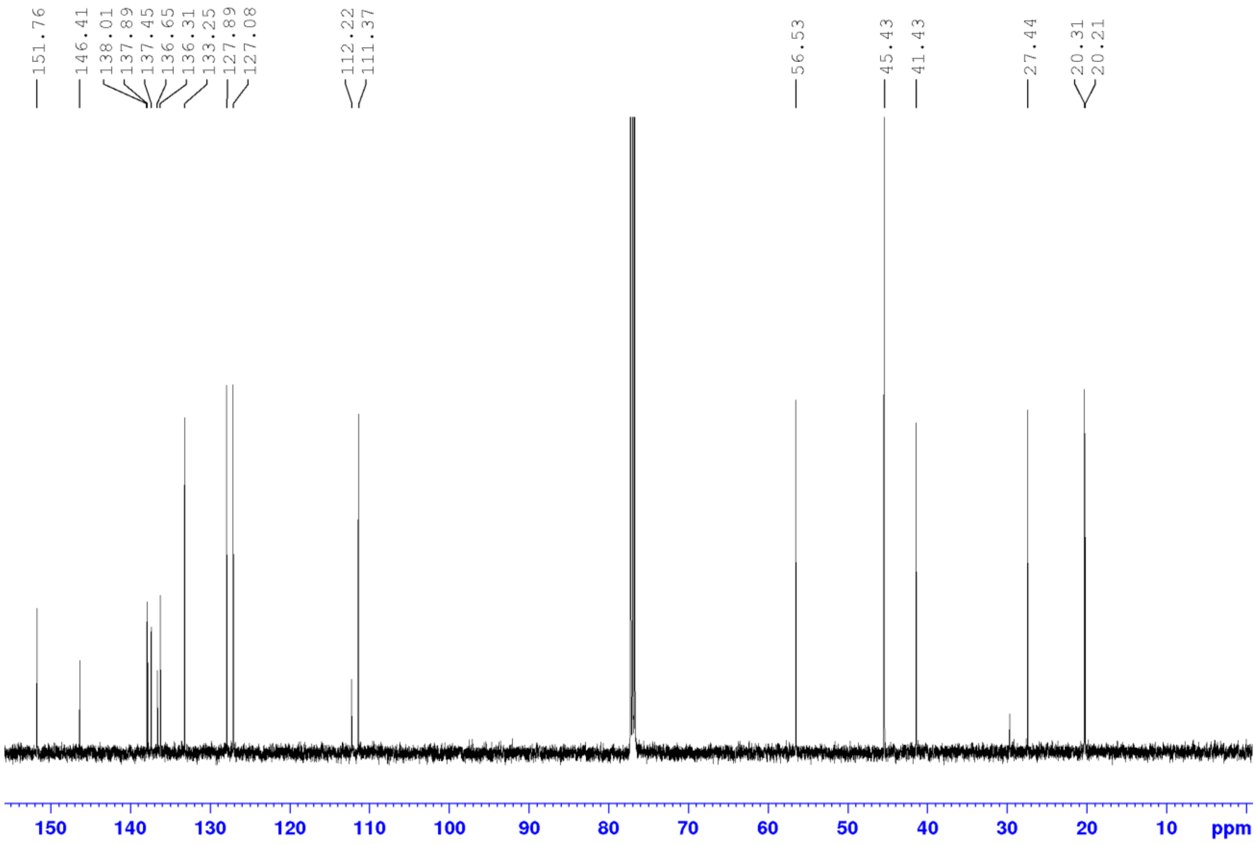

^{1}H (500 MHz, $CDCl_3$) and ^{13}C (125 MHz, $CDCl_3$) NMR Spectra of **8b**.

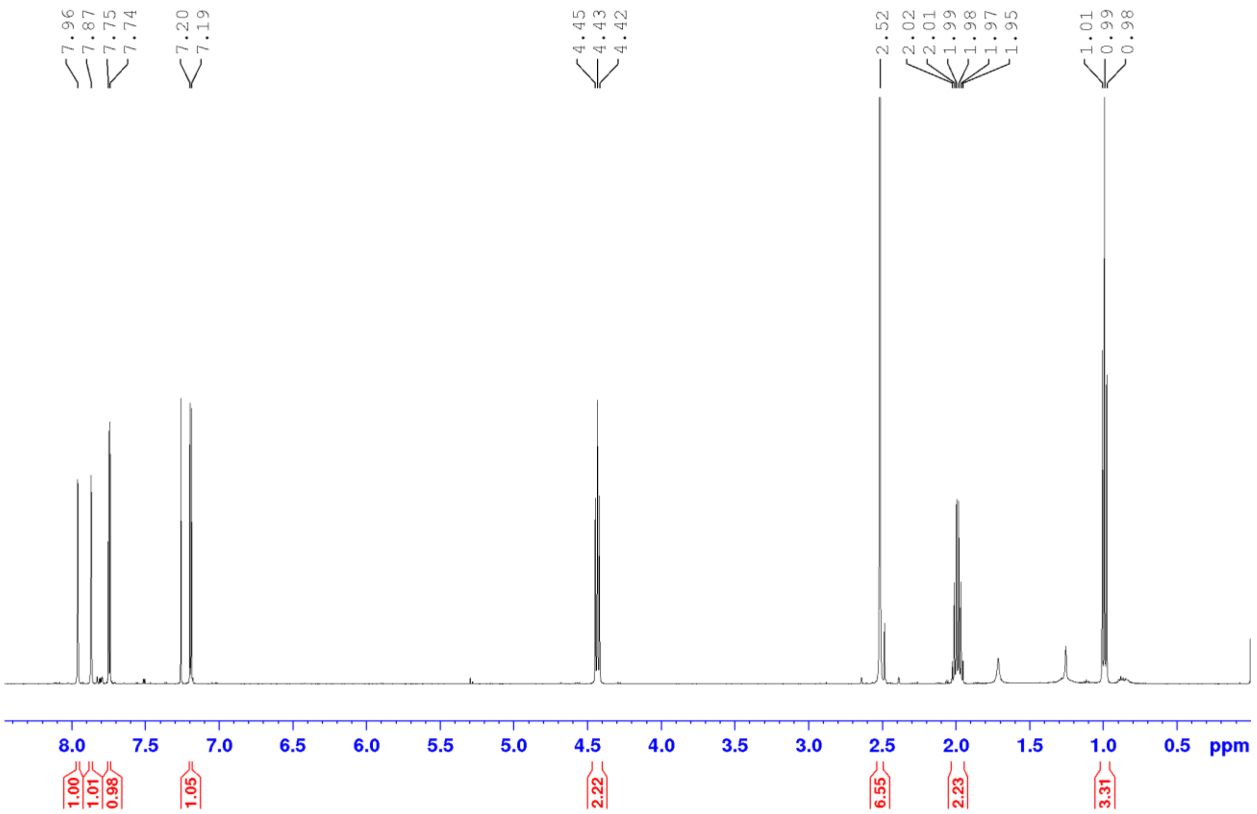

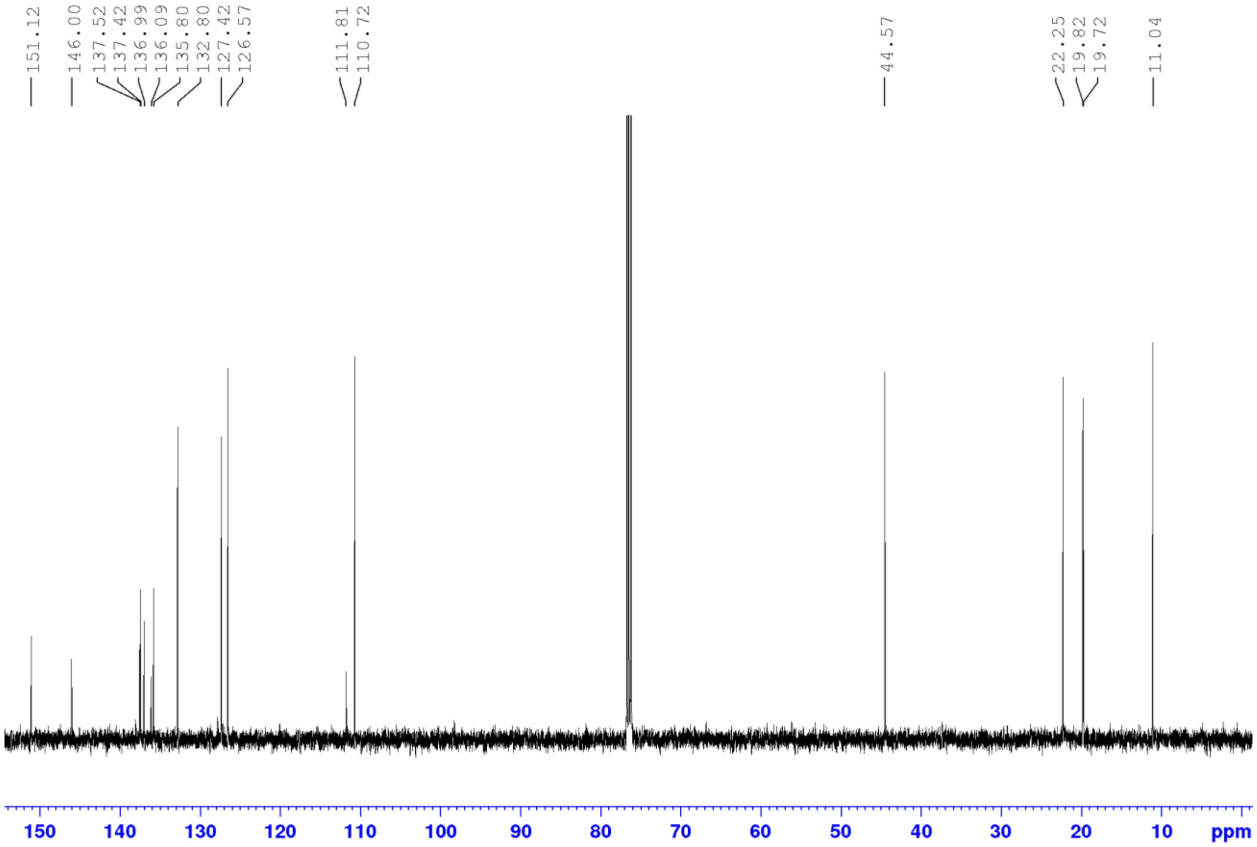

^{1}H (500 MHz, CDCl$_3$) and ^{13}C (125 MHz, CDCl$_3$) NMR Spectra of **9b**.

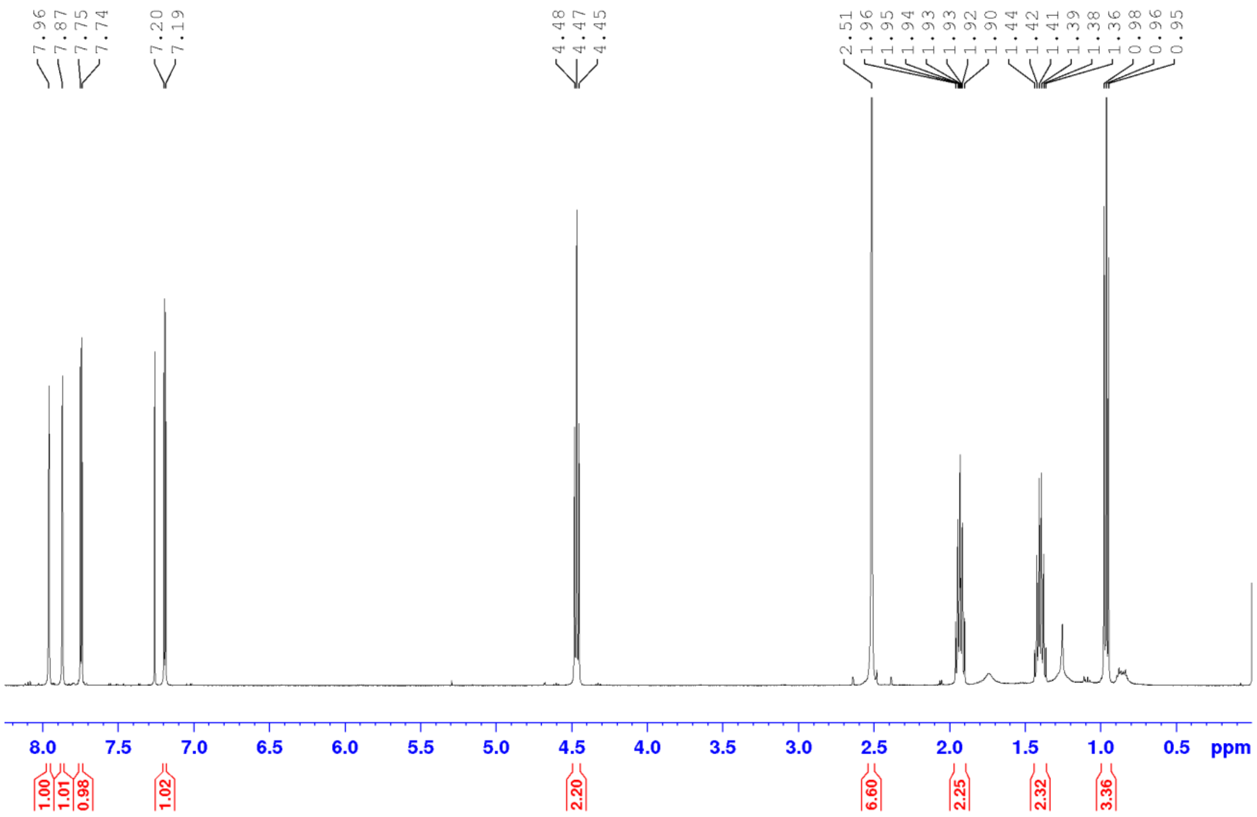

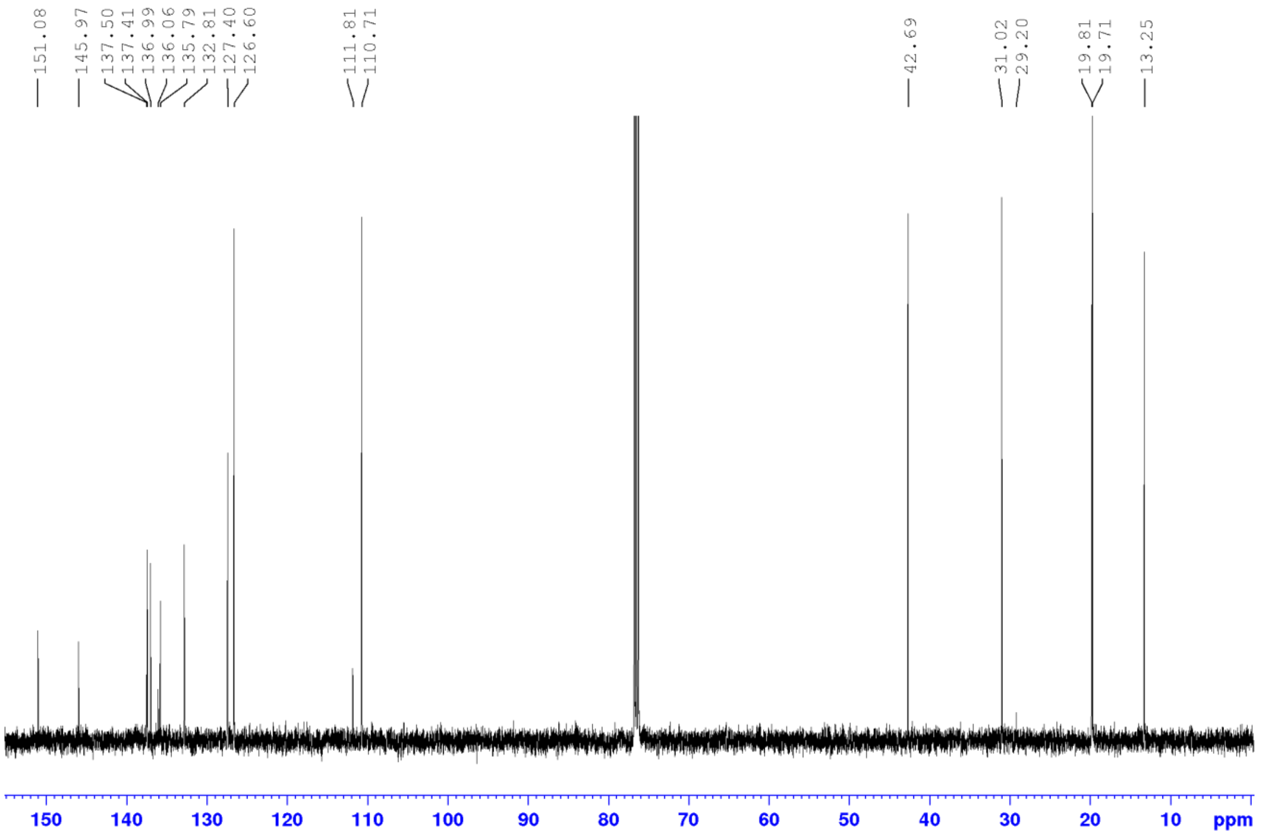

^{1}H (500 MHz, $CDCl_3$) and ^{13}C (125 MHz, $CDCl_3$) NMR Spectra of **10b**.

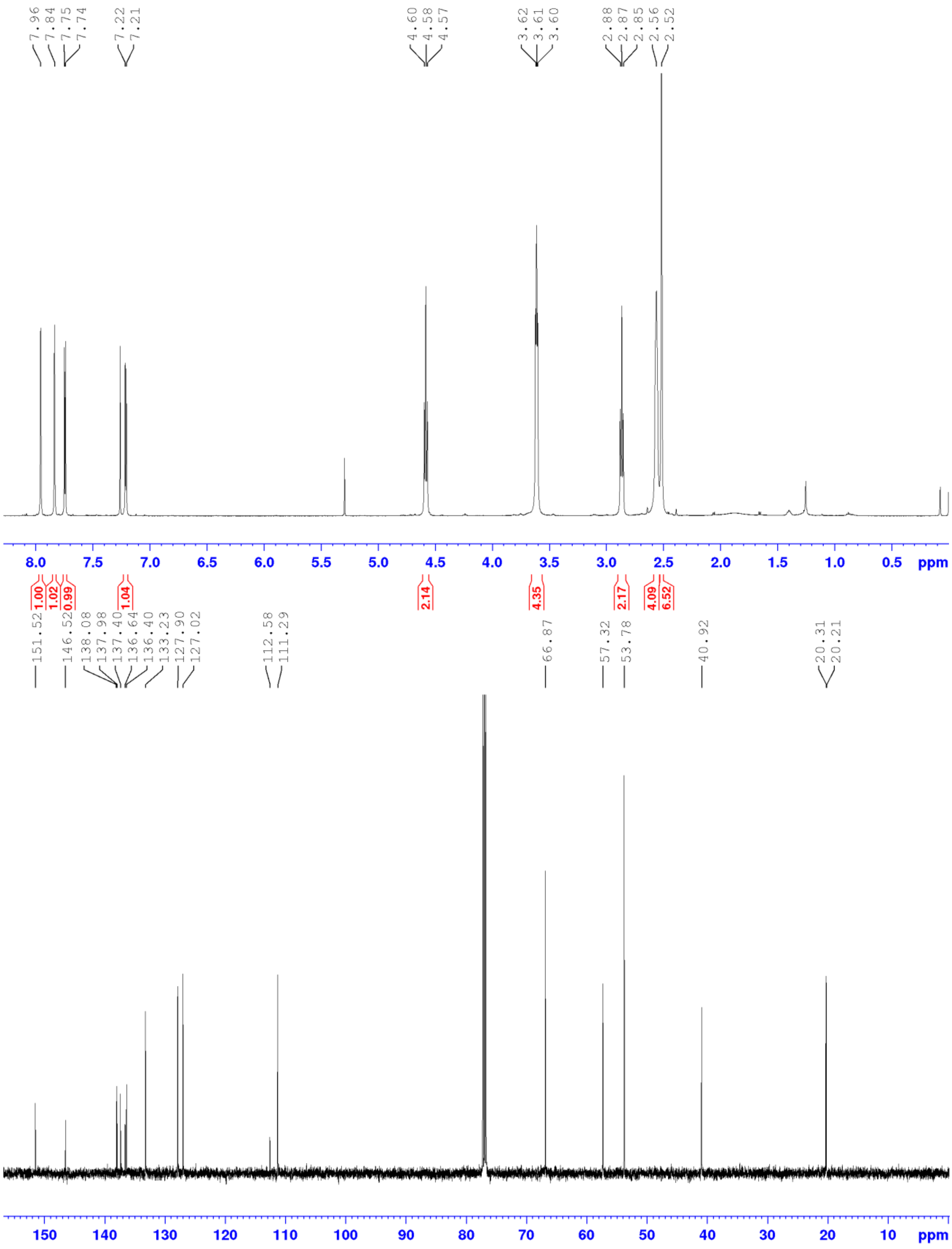

^{1}H (500 MHz, $CDCl_3$) and ^{13}C (125 MHz, $CDCl_3$) NMR Spectra of **11b**.
